# Does High-Dose Antimicrobial Chemotherapy Prevent the Evolution of Resistance?

**DOI:** 10.1371/journal.pcbi.1004689

**Published:** 2016-01-28

**Authors:** Troy Day, Andrew F. Read

**Affiliations:** 1 Department of Mathematics and Statistics, Jeffery Hall, Queen’s University, Kingston, Ontario, Canada; 2 Department of Biology, Queen’s University, Kingston, Ontario, Canada; 3 The Fogarty International Center, National Institutes of Health, Bethesda, Maryland, United States of America; 4 Center for Infectious Disease Dynamics, Departments of Biology and Entomology, The Pennsylvania State University, University Park, Pennsylvania, United States of America; Ecole Polytechnique Federale de Lausanne, SWITZERLAND

## Abstract

High-dose chemotherapy has long been advocated as a means of controlling drug resistance in infectious diseases but recent empirical studies have begun to challenge this view. We develop a very general framework for modeling and understanding resistance emergence based on principles from evolutionary biology. We use this framework to show how high-dose chemotherapy engenders opposing evolutionary processes involving the mutational input of resistant strains and their release from ecological competition. Whether such therapy provides the best approach for controlling resistance therefore depends on the relative strengths of these processes. These opposing processes typically lead to a unimodal relationship between drug pressure and resistance emergence. As a result, the optimal drug dose lies at either end of the therapeutic window of clinically acceptable concentrations. We illustrate our findings with a simple model that shows how a seemingly minor change in parameter values can alter the outcome from one where high-dose chemotherapy is optimal to one where using the smallest clinically effective dose is best. A review of the available empirical evidence provides broad support for these general conclusions. Our analysis opens up treatment options not currently considered as resistance management strategies, and it also simplifies the experiments required to determine the drug doses which best retard resistance emergence in patients.

## Author Summary

The evolution of antimicrobial resistant pathogens threatens much of modern medicine. For over one hundred years, the advice has been to ‘hit hard’, in the belief that high doses of antimicrobials best contain resistance evolution. We argue that nothing in evolutionary theory supports this as a good rule of thumb in the situations that challenge medicine. We show instead that the only generality is to either use the highest tolerable drug dose or the lowest clinically effective dose; that is, one of the two edges of the therapeutic window. This approach suggests treatment options not currently considered, and simplifies the experiments required to identify the dose that best retards resistance evolution.

## Introduction

Antimicrobial resistance is one of greatest challenges faced by modern medicine. There is a widely held view that the evolutionary emergence of drug resistance is best slowed by using high doses of drugs to eliminate pathogens as early and quickly as possible. This view, first expounded by Ehrlich [1] (‘hit hard’) and later Fleming [2] (‘if you use penicillin, use enough’), is today encapsulated in the advice to administer ‘the highest tolerated antibiotic dose’ [3, 4]. The rationale is two-fold. First, a high concentration of drug will eliminate drug-sensitive microbes quickly and thereby limit the appearance of resistant strains. Second, a high concentration of drug will also eliminate strains that have some partial resistance, provided the concentration is above the so-called mutant prevention concentration (MPC) [5–12].

This is an intuitively appealing idea, but several authors have recently questioned whether high-dose chemotherapy is, as a generality, defensible in terms of evolutionary theory [13–16]. This is because the use of extreme chemical force comes at the cost of maximizing the selective advantage of the very pathogens that we fear most; namely, those which cannot be eradicated by safely administered doses of drug. Some experimental studies have also shown that lighter-touch chemotherapy not only better prevents the emergence of resistance but it restores host health just as well as high-dose chemotherapy [15–17].

Here we use principles from evolutionary biology to provide a general and comprehensive theoretical framework for studying the effects of different drug treatment strategies. The analysis shows that high-dose chemotherapy gives rise to opposing evolutionary processes. As a result, the optimal therapy for controlling resistance depends on the relative strengths of these processes. High-dose therapy can, in some circumstances, retard resistance emergence but evolutionary theory provides no support for using this strategy as a general rule of thumb, nor does it provide support for focussing on the MPC as a general approach for resistance prevention. More broadly we find that the opposing evolutionary processes lead to a unimodal relationship between drug concentration and resistance emergence. Therefore the optimal strategy is to use either the largest tolerable dose or the smallest clinically effective dose. We illustrate these general points with some simple models that show how a seemingly minor change in parameter values can alter the outcome from one where high-dose chemotherapy is optimal to one where using the smallest clinically effective dose is best. A review of the empirical evidence provides broad support for these conclusions.

## Methods

Determining a patient treatment regimen involves choosing an antimicrobial drug (or drugs) and determining the frequency, timing, and duration of administration. The impact of each of these on resistance emergence has been discussed elsewhere [9, 18]. Here we focus solely on drug concentration because it has historically been the factor most often discussed and because it is the source of recent controversy [10, 12–14, 16]. We seek to understand how the probability of resistance emergence changes as a function of drug concentration.

For simplicity we assume that drug concentration is maintained at a constant level during treatment and refer to this concentration as ‘dose’. This assumption is not meant to be realistic but it serves as a useful tool for gaining a better understanding of how drug resistance evolves. After laying the groundwork for this simple case we show in the Supporting Information that allowing for more realistic pharmacokinetics does not alter our qualitative conclusions.

Drug resistance is a matter of degree, with different genotypes having different levels of resistance (measured, for example, as the minimum inhibitory concentration, MIC). Our main focus is on what we call high-level resistance (HLR). This will be defined precisely below but for the moment it can be thought of as resistance that is high enough to render the drug ineffective (so that its use is abandoned). We begin by supposing that the HLR strain is one mutational step away from the wild type but we relax this assumption in the Supporting Information.

Why is it that resistant strains reach appreciable densities in infected patients only once drug treatment is employed? The prevailing view is that there is a cost of resistance in the absence of the drug but that this cost is compensated for by resistance in the presence of the drug. It is not the presence of the drug *per se* that provides this compensation; rather, it is the removal of the wild type by the drug that does so [13, 19]. This implies that the presence of the wild type competitively suppresses the resistant strain and that drugs result in the spread of such strains because they remove this competitive suppression (a process called ‘competitive release’; [19]).

To formalize these ideas, consider an infection in the absence of treatment. The wild type pathogen enters the host and begins to replicate. As it does so, it consumes resources and stimulates an immune response. We use *P*(*t*) to denote the density of the wild type and *X*(*t*) to denote a vector of within-host state variables (e.g., density of immune system components, resources, etc). Without loss of generality we suppose that the vector *X* is defined in such a way that pathogen replication causes its components to decrease. For example, if a component of the state vector represents some element of an immune response, then we can define this component of *X*(*t*) as the inverse of this immune cell density. This decrease in *X*, in turn, makes the within-host environment less favorable for pathogen replication. If *X* is suppressed enough, the net replication rate of the wild type will reach zero. Thus *X* can be viewed as the quality of the within-host environment from the standpoint of pathogen replication.

As the wild type replicates it gives rise to the HLR strain through mutation and the initial infection might include some HLR pathogens as well. But the HLR strain is assumed to bear some metabolic or replicative cost, meaning that it is unable to increase in density once the wild type has become established. Mechanistically this is because the wild type suppresses the host state, *X*, below the minimum value required for a net positive replication by the HLR strain [19]. Thus, we ignore the effect of the HLR strain when modeling the joint dynamics of *P*(*t*) and *X*(*t*) in the absence of treatment (see Appendix 1 in S1 Text for details).

At some point (e.g., the onset of symptoms) drug treatment is introduced. Provided the dosage is high enough the wild type will be driven to extinction. We use *c* to denote the (constant) concentration of the drug in the patient. We distinguish between *theoretically possible* versus *feasible* doses. Theoretically possible doses are those that can be applied *in vitro*. Feasible doses are those that can, in practice, be used *in vivo*. There will be a smallest clinically effective dose that places a lower bound on the feasible values of *c* (denoted *c*_*L*_) and a maximum tolerable dose because of toxicity (denoted *c*_*U*_). The dose range between these bounds is called the therapeutic window [20].

Once treatment has begun, we use *p*(*t*;*c*) and *x*(*t*;*c*) to denote the density of the wild type strain and the within-host state. This notation reflects the fact that different dosages (i.e., concentrations) will give rise to different trajectories of *p* and *x* during the remainder of the infection. We model the dynamics of *p* and *x* deterministically during this phase.

As the wild type is driven to extinction it will continue to give rise to HLR microbes through mutation. The mutation rate is given by a function *λ*[*p*(*t*; *c*), *c*] that is increasing in *p* and decreasing in *c*. We suppose that lim_*c* → ∞_ *λ*[*p*, *c*] = 0 because a high enough drug concentration will completely suppress wild type replication and thus mutation. Any HLR microbes that are present during treatment will no longer be destined to rarity because they will be released from competitive suppression [19]. We use *π*[*x*(*t*; *c*), *c*] to denote the probability of escaping initial extinction when rare. The function *π* is increasing in *x* because it is through this state that the HLR strain has been competitively suppressed [19]. And *π* is decreasing in *c* with lim_*c* → ∞_ *π*[*x*, *c*] = 0 because a high enough dose will also suppress even the HLR strain.

We can now provide a precise definition of high-level resistance (HLR). Although lim_*c* → ∞_ *π*[*x*, *c*] = 0, the concentration at which this limit is reached can lie outside the therapeutic window [*c*_*L*_, *c*_*U*_]. We define HLR to mean that *π*[*x*, *c*] is very nearly equal to *π*[*x*, 0] over the therapeutic window. Biologically this means that, in terms of clinically acceptable doses, significant suppression of HLR is not possible. We focus on HLR because, for genotypes that do not satisfy this property, there is then no resistance problem to begin (since one can always use a high enough dose to remove all pathogens). For instance, there is evidently no resistance problem in HIV and Hepatitis C when patients are fully compliant with recommended combination therapy regimens [21]. That is because at clinically acceptable doses of those combination therapies, mutations conferring HLR do not arise. We are here interested in cases in which significant suppression of HLR is not possible even at the upper end of the therapeutic window.

With the above formalism, we focus on resistance emergence, defined as the replication of resistant microbes to a high enough density within a patient to cause symptoms and/or to be transmitted [19]. In the analytical part of our results this is equivalent to the resistant strain not being lost by chance while rare.

With the above assumptions the host can be viewed as being in one of two possible states at any point in time during the infection: (i) resistance has emerged (i.e., a resistant strain has appeared and escaped), or (ii) resistance has not emerged. We model the transition between these two states as an inhomogeneous birth process. Appendix 1 in S1 Text then shows that the probability of resistance emergence is approximately equal to 1−*e* ^−*H*(*c*)^ where

$$\begin{array}{ccc} H(c) & = & D(c)+S(c) \end{array}$$ (1)

and

$$\begin{array}{c} D\left(c\right)=\int_{0}^{a}\lambda \left[p\left(s;c\right),c\right]\;\pi \left[x\left(s;c\right),c\right]\text{d}s \end{array}$$ (2)

$$\begin{array}{c} S\left(c\right)=-n\ln\left(1-\pi [x(0;c),c]\right) \end{array}$$ (3)

We refer to *H*(*c*) as the resistance ‘hazard’, and *a* is the duration of treatment with *s* = 0 corresponding to the start of treatment. The quantity *D*(*c*) is the *de novo* hazard—it is the hazard due to resistant strains that appear *de novo* during treatment. It is comprised of the integral of the product of *λ*[*p*(*s*; *c*), *c*], the rate at which resistant mutants appear at time *s* after the start of treatment, and *π*[*x*(*s*; *c*), *c*], the probability of escape of any such mutant. The quantity *S*(*c*) is the standing hazard—it is the hazard due to a standing population of *n* resistant microbes that are already present at the beginning of treatment (see Appendix 1 in S1 Text). To minimize the probability of resistance emergence we therefore want to minimize the hazard *H*(*c*), subject to the constraint that the dosage *c* falls within the therapeutic window [*c*_*L*_, *c*_*U*_].

## Results

To determine how high-dose chemotherapy affects the probability of resistance emergence we determine how *H*(*c*) changes as drug dosage *c* increases. Differentiating expression Eq (1) with respect to *c* we obtain

dHdc=∫0aπ∂λ∂p∂p∂c+∂λ∂cds︷mutation∫0aπ∂λ∂p∂p∂c+∂λ∂cds+∫0aλ∇xπ·xc+∂π∂cds︸de novo hazard∫0aλ∇xπ·xc︸+∂π∂cds+n1-π∇xπ0·xc0︸+∂π0∂c︸standing hazard︷replication (4)

where *π* ^0^ = *π*[*x*(0; *c*), *c*], *x* ^0^ = *x*(0; *c*), and subscripts denote differentiation. Eq (4) is partitioned in two different ways to better illustrate the effect of increasing dose. The first is a partitioning of its effect on mutation and replication. The second is a partitioning of its effect on the *de novo* and standing hazards. We have also indicated the terms that represent competitive release by underlining them (as explained below).

The first term in Eq (4) represents the change in *de novo* mutation towards the HLR strain that results from an increase in dose. The term (∂*λ*/∂*p*)(∂*p*/∂*c*) is the change in mutation rate, mediated through a change in wild type density; ∂*λ*/∂*p* specifies how mutation rate changes with an increase in the wild type density *p* (positive) while ∂*p*/∂*c* specifies how the wild type density changes with an increase in dose (typically negative for much of the duration of treatment). Thus the product, when integrated over the duration of treatment, is expected to be negative. The term ∂*λ*/∂*c* is the change in mutation rate that occurs directly as a result of an increased dose (e.g., the direct suppression of wild type replication, which suppresses mutation). This, is expected to be non-positive in the simplest cases and is usually taken as such by proponents of high-dose chemotherapy. Therefore *high-dose chemotherapy decreases the rate at which HLR mutations arise during treatment*. Note, however, that if the drug itself causes a higher mutation rate [22], then it is possible for an increased dose to increase the rate at which resistance appears. The same would be true if resistance was mediated by an increased physiological expression of efflux pumps or processes like antibiotic metabolism. In any of these situations the use of high-dose chemotherapy would then be even more risky from the standpoint of resistance emergence.

The second term in Eq (4) represents replication of HLR strains once they have appeared *de novo* during the course of treatment. The term ∇_*x*_ *π* ⋅ *x*_*c*_ is the indirect increase in escape probability, mediated through the effect of within-host state, *x*. Specifically, *x*_*c*_ is a vector whose elements give the change in each state variable arising from an increased dosage (through the removal of the wild type). These elements are typically expected to be positive for much of the duration of treatment because an increase in dose causes an increased rebound of the within-host state through a heightened removal of wild type microbes. The quantity ∇_*x*_ *π* is the gradient of the escape probability with respect to host state *x*, and its components are expected to be positive (higher state leads to a greater probability of escape). The integral of the dot product ∇_*x*_ *π* ⋅ *x*_*c*_ is therefore the competitive release of the HLR strain in terms of *de novo* hazard [19]. This will typically be positive. The term ∂*π*/∂*c* is the direct change in escape probability of de novo mutants as a result of an increase in dosage (i.e., the extent to which the drug suppresses even the HLR strain). This term is negative at all times during treatment but, by the definition of HLR, this is small. Therefore, *high-dose chemotherapy increases the replication of any HLR mutants that arise de novo during treatment*.

Finally, the third term in Eq (4) represents the replication of any HLR strains that are already present at the start of treatment. The term $\frac{n}{1-\pi }\;\left(\nabla_{x}\pi^{0}\cdot x_{c}^{0}\right)$ is the indirect effect of dose on standing hazard, where *n* is the number of resistant pathogens present at the start of treatment. The quantity $x_{c}^{0}$ is again a vector whose elements give the change in state arising from increased dosage (through the removal of the wild type). The components of this are typically expected to be positive because an increase in dose causes a rebound in the within-host state. ∇_*x*_ *π* ^0^ is the gradient of the escape probability with respect to state, and its components are expected to be positive (higher state leads to greater probability of escape). The dot product of the two, $\nabla_{x}\pi^{0}\cdot x_{c}^{0}$, is therefore the competitive release of the HLR strain in terms of standing hazard [19]. This will typically be positive. The term $\frac{n}{1-\pi }\frac{\partial \pi^{0}}{\partial c}$ is the direct change in escape probability of pre-existing mutants as a result of an increase in dosage (i.e., the extent to which the drug suppresses even these HLR mutants) and is negative. Again, however, by the definition of HLR, this will be small and therefore *high-dose chemotherapy increases the replication of any HLR mutants that are present at the start of treatment*. Appendix 2 in S1 Text shows that the same set of qualitative factors arise if there are strains with intermediate resistance as well.

The above results provide a mathematical formalization of earlier verbal arguments questioning the general wisdom of using high-dose chemotherapy as a means of controlling resistance emergence [13, 16]. Advocates of the conventional heavy dose strategy tend to emphasize how high-dose chemotherapy can reduce mutational input and potentially even suppress the replication of resistant strains (the terms in Eq 4 that are not underlined). However, high-dose chemotherapy leads to competitive release and thus greater replication of any resistant strains that are present (the underlined terms in Eq 4). Eq (4) shows that it is the relative balance among these opposing processes that determines whether high-dose chemotherapy is the optimal approach. We will present a specific numerical example shortly that illustrates these points, but first we draw two more general conclusions from the theory.

### Intermediate doses yield the largest hazard and thus the greatest likelihood of resistance emergence across all theoretically feasible doses

The opposing evolutionary processes explained above are the reason why intermediate doses yield the largest hazard [16]. First note that the functions *λ* and *π* will typically be such that *D*(0) ≈ 0. In other words, the HLR strain does not emerge *de novo* within infected individuals if they are not receiving treatment. Mechanistically, this is because any resistant strains that appear tend to be competitively suppressed by the wild type strain [19]. Although, *S*(0) need not be zero (see S2 Fig), the rate of change of *S*(*c*) with respect to *c* (i.e., the third term in Eq 4) is positive at *c* = 0. Therefore the maximum hazard cannot occur at *c* = 0.

Second, for large enough doses we have *π*[*x*(*s*; *c*), *c*] ≈ 0 for all *s* because such extreme concentrations will prevent replication of even the HLR strain. This makes both the *de novo* hazard *D*(*c*) and the standing hazard *S*(*c*) zero. Furthermore, for large enough *c* we also have *λ*[*p*(*s*; *c*), *c*]≈0 for all *s* as well if HLR can arise only during wild type replication, because such extreme concentrations prevent all replication of the wild type. This is an additional factor making the *de novo* hazard *D*(*c*) decline to zero for large *c*. Therefore lim_*c* → ∞_ *H*(*c*) = 0 and so the maximum hazard cannot occur for large values of *c* either [16]. Thus, the maximum hazard must occur for an intermediate drug dosage. Although this prediction is superficially similar to that of the mutant selection window hypothesis [5–9], there are important differences between the two as will be elaborated upon in the discussion.

### The optimal dose is either the maximum tolerable dose or minimum clinically effective dose

**Fig 1:**
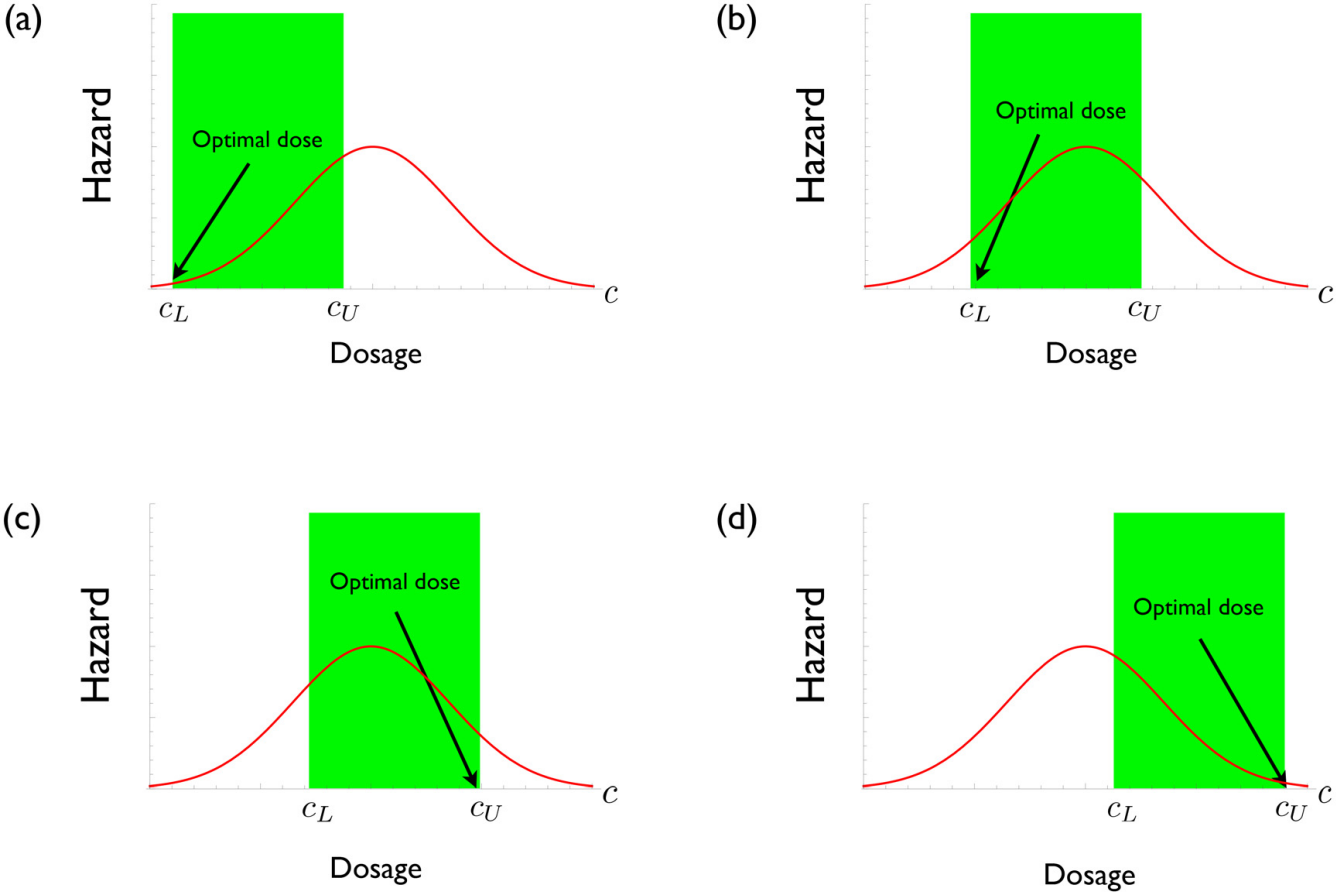
Hypothetical plots of resistance hazard *H*(*c*) as a function of drug concentration *c*. The lowest effective dose and the highest tolerable dose are denoted by *c*_*L*_ and *c*_*U*_ respectively. The therapeutic window is shown in green. (a) and (b) drug concentration with the smallest hazard is the lowest effective dose. (c) and (d) drug concentration with the smallest hazard is the highest tolerable dose.

We have seen that the maximum hazard occurs for an intermediate dose. Although in principle the hazard function might be quite complex, in practice our models have never produced anything other than a unimodal relationship between *H*(*c*) and *c* (i.e., a single maximum). Furthermore, because the maximum hazard must always occur at an intermediate dose, even if the theoretical hazard curve is multimodal the existence of error in drug delivery and other sources of noise will tend to make the empirical hazard curve unimodal (Appendix 1 in S1 Text). As will be seen in the Discussion, an extensive body of empirical work also shows that measured hazard curves always appear to be unimodal. As a result, the drug dose which best reduces the probability of resistance emergence is always at one of the two extremes of the therapeutic window. This means that it is best to use either the smallest clinically effective dose or the largest tolerable dose depending on the situation, but never anything in between (Fig 1).

### A specific example

To illustrate the general theory we now consider an explicit model for the within-host dynamics of infection and resistance. We model an acute infection in which the pathogen elicits an immune response that can clear the infection. Treatment is nevertheless called for because, by reducing the pathogen load, it reduces morbidity and mortality (see Appendix 3 in S1 Text for details).

**Fig 2:**
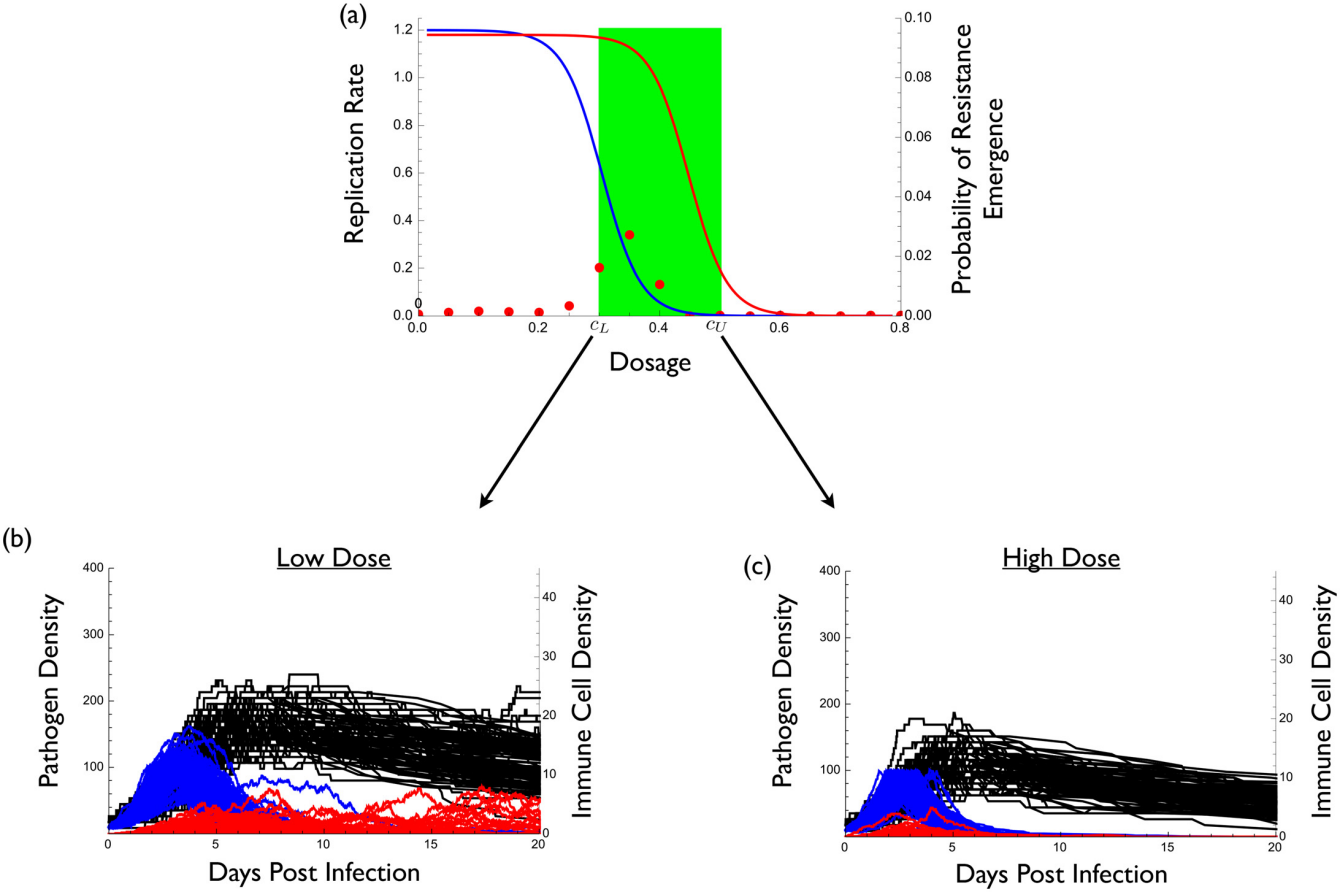
Example where conventional strategy of high-dose chemotherapy best prevents the emergence of resistance. (a) The dose-response curves for the wild type in blue (*r*(*c*) = 0.6(1−tanh(15(*c*−0.3)))) and the resistant strain in red (*r*_*m*_(*c*) = 0.59(1−tanh(15(*c*−0.45)))) as well as the therapeutic window in green. Red dots indicate the probability of resistance emergence. Probability of resistance emergence is defined as the fraction of 5000 simulations for which resistance reached a density of at least 100 (and thus caused disease).(b) and (c) wild type density (blue), resistant density (red), and immune molecule density (black) during infection for 1000 representative realizations of a stochastic implementation of the model. (b) treatment at the smallest effective dose *c*_*L*_, (c) treatment at the maximum tolerable dose *c*_*U*_. Parameter values are *P*(0) = 10, *P*_*m*_(0) = 0, *I*(0) = 2, *α* = 0.05, *δ* = 0.05, *κ* = 0.075, *μ* = 10^−2^, and *γ* = 0.01.

We begin by considering a situation in which the maximum tolerable drug concentration *c*_*U*_ causes significant suppression of the resistant strain (Fig 2a). We stress however that if this were true then, by definition, the resistant strain is not really HLR and thus there really is no resistance problem to begin with. We include this extreme example as a benchmark against which comparisons can be made.

Not surprisingly, under these conditions a large dose is most effective at preventing resistance (compare Fig 2b with 2c). This is a situation in which the conventional ‘hit hard’ strategy is best. Modern treatment of HIV is an example of this. With combination therapy and good patient compliance, it is evidently possible to completely prevent virus replication and thus the emergence of resistance [18].

**Fig 3:**
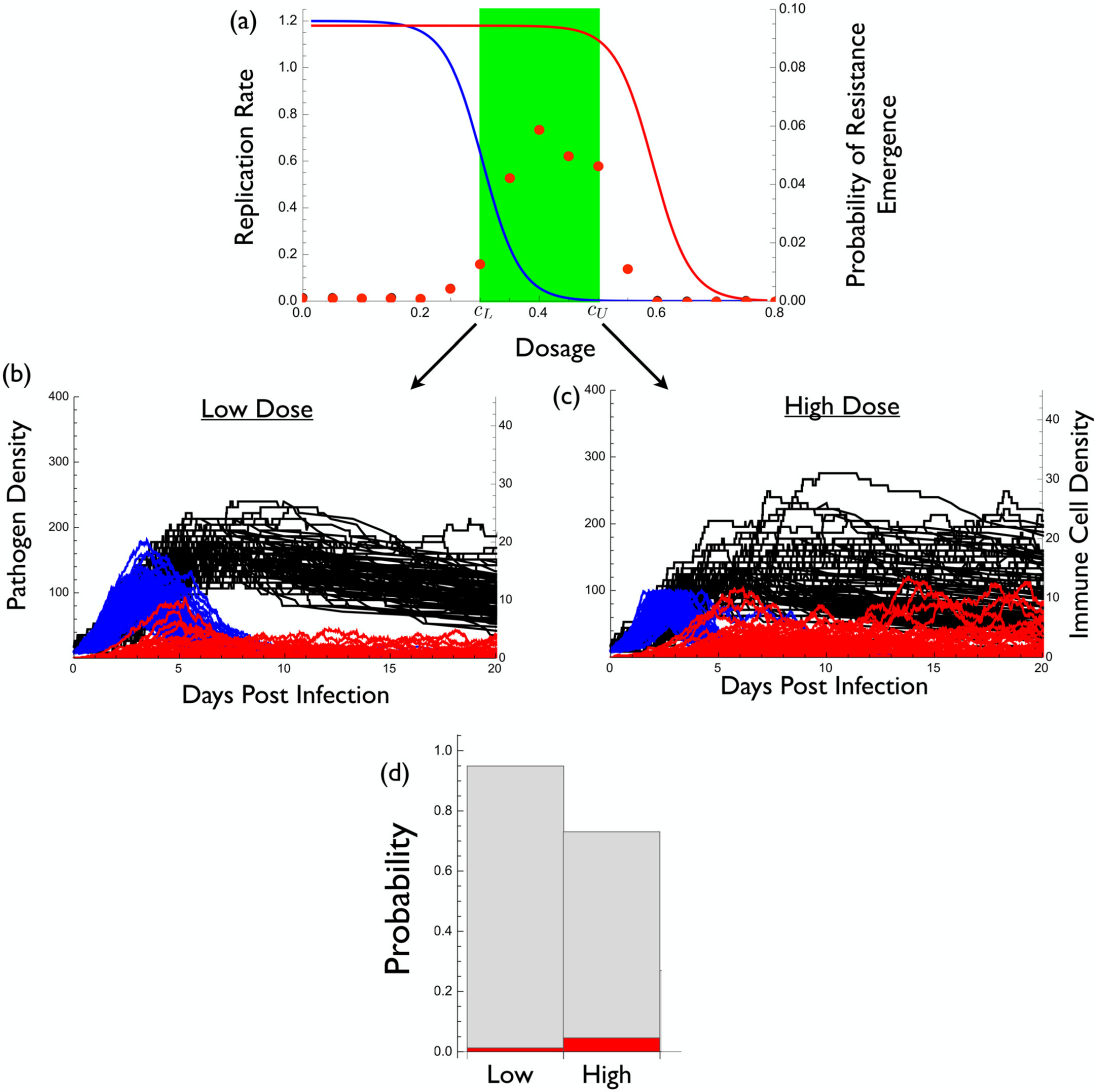
Example where low-dose strategy best prevents the emergence of resistance. (a) The dose-response curves for the wild type in blue (*r*(*c*) = 0.6(1−tanh(15(*c*−0.3)))) and the resistant strain in red (*r*_*m*_(*c*) = 0.59(1−tanh(15(*c*−0.6)))) as well as the therapeutic window in green. Red dots indicate the probability of resistance emergence. Probability of resistance emergence is defined as the fraction of 5000 simulations for which resistance reached a density of at least 100 (and thus caused disease).(b) and (c) wild type density (blue), resistant density (red), and immune molecule density (black) during infection for 1000 representative realizations of a stochastic implementation of the model. (b) treatment at the smallest effective dose *c*_*L*_, (c) treatment at the maximum tolerable dose *c*_*U*_. (d) The probability that a resistant strain appears by mutation is indicated by grey bars for low and high dose. The probability of resistance emergence is indicated by the height of the red bars for these cases. The probability of resistance emergence, given a resistant strain appeared by mutation, can be interpreted as the ratio of the red to grey bars. Parameter values are *P*(0) = 10, *P*_*m*_(0) = 0, *I*(0) = 2, *α* = 0.05, *δ* = 0.05, *κ* = 0.075, *μ* = 10^−2^, and *γ* = 0.01.

Now suppose that the maximum tolerable drug concentration *c*_*U*_ is not sufficient to directly suppress the resistant strain (Fig 3a). In this case the only difference from Fig 2 is a change in the resistant strain’s dose-response curve. Now there really is a potential resistance problem in the sense that, from a clinical standpoint, the drug is largely ineffective against the resistant strain.

Under these conditions we see that a small dose is more effective at preventing resistance emergence than a large dose (compare Fig 3b with 3c). This is a situation in which the conventional or orthodox ‘hit hard’ strategy is not optimal.

Eq (4) provides insight into these contrasting results. The only difference between the models underlying Figs 2 and 3 is that ∂*π*/∂*c* and ∂*π* ^0^/∂*c* are both negative for Fig 2 whereas they are nearly zero for Fig 3 (that is, at tolerable doses, the drug has negligible effects on resistant mutants). As a result, the negative terms in Eq (4) outweigh the positive terms for Fig 2 whereas the opposite is true for Fig 3.

These results appear to contradict those of a recent study by Ankomah and Levin [12]. Although their model is more complex than that used here, Eq (4) and its extensions in S1 Text show that such additional complexity does not affect our qualitative conclusions. Ankomah and Levin [12] defined resistance evolution in two different ways: (i) the probability of emergence, and (ii) the time to clearance of infection. For the sake of comparison, here we focus on the probability of emergence. Ankomah and Levin [12] defined emergence as the appearance of a single resistant microbe. As such their emergence is really a measure of the occurrence of resistance mutations rather than emergence *per se*.

In comparison, we consider emergence to have occurred only once the resistant strain reaches clinically significant levels; namely, a density high enough to cause symptoms or to be transmitted. There are two process that must occur for *de novo* resistant strains to reach clinically relevant densities. First, the resistant strain must appear by mutation, and both our results (Fig 3d) and those of Ankomah and Levin [12] show that a high dose better reduces the probability that resistance mutations occur (this can also be seen in Eq 4). Second, the resistant strain must replicate to clinically significant levels. Ankomah and Levin [12] did not account for this effect and our results show that a high concentration is worse for controlling the replication of resistant microbes *given a resistant strain has appeared* (Fig 3d). This is because higher doses maximally reduce competitive suppression. In Fig 3 the latter effect overwhelms the former, making low-dose treatment better. In Fig 2 these opposing processes are also acting but in that case the drug’s effect on controlling mutation outweighs its effect on increasing the replication of such mutants once they appear.

**Fig 4:**
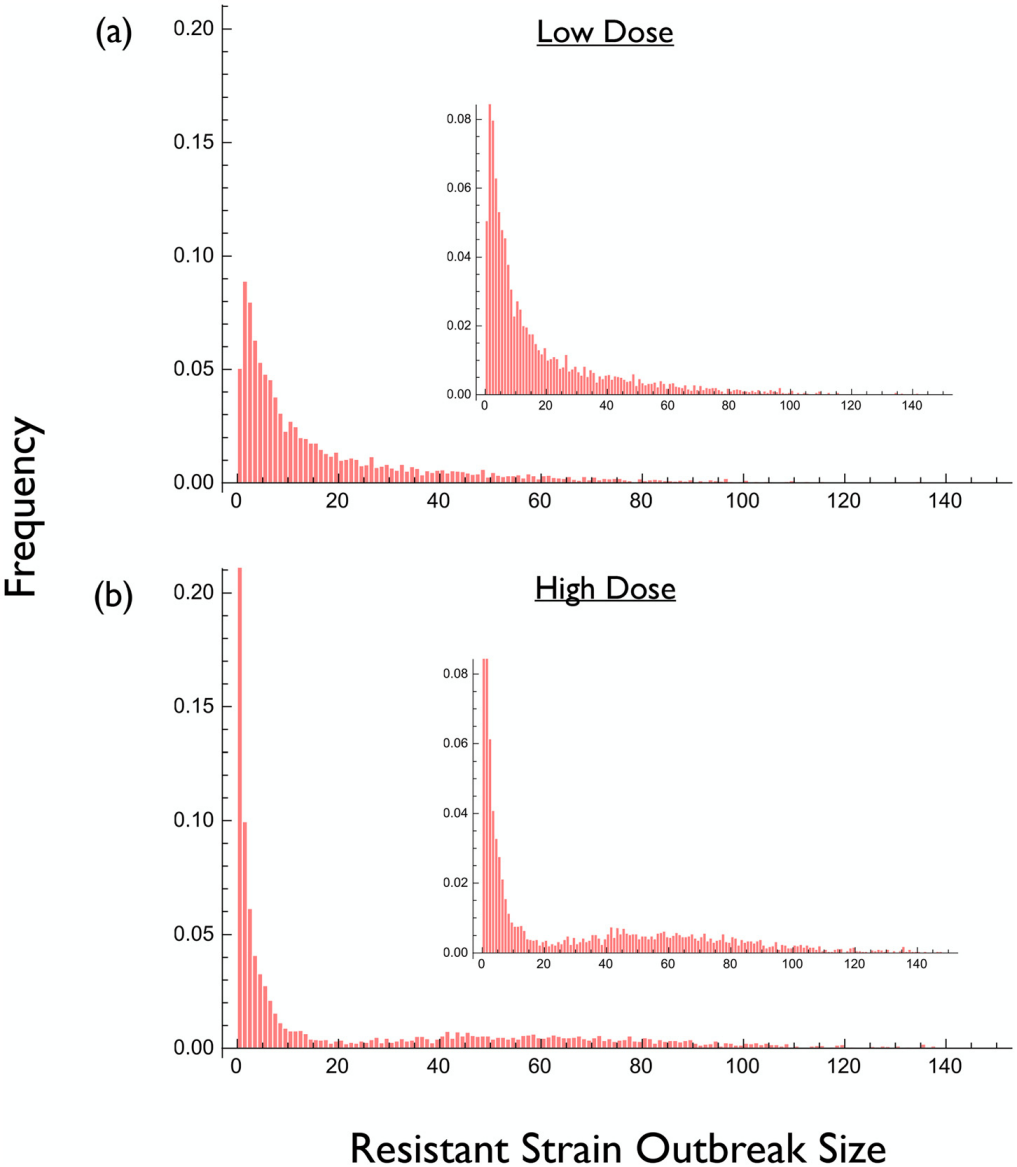
Frequency distribution of resistant strain outbreak sizes for the simulation underlying Fig 3. Each distribution is based on 5000 realizations of a stochastic implementation of the model. (a) Low drug dose. (b) High drug dose. Insets show the same distribution on a different vertical scale.

More generally, Fig 4 illustrates the relationship between drug concentration and the maximum size of the resistant population during treatment, for the model underlying Fig 3. In this example a high concentration tends to result in relatively few outbreaks of the resistant strain but when they occur they are very large. Conversely, a low concentration tends to result in a greater number of outbreaks of the resistant strain but when they occur they are usually too small to be clinically significant.

One can also examine other metrics like duration of infection, total resistant strain load during treatment, likelihood of resistant strain transmission, etc. but the above results are sufficient to illustrate that no single, general, result emerges. Whether a high or low dose is best for managing resistance will depend on the specific context (i.e., the parameter values) as well as the metric used for quantifying resistance emergence. In Appendices 3–6 of S1 Text we consider cases where there is pre-existing resistance at the start of infection, strains with intermediate resistance, other measures of drug dosing and resistance emergence, a model of chronic infection based on resource competition, and more general pharmacokinetics. None of these factors alters the general finding that the optimal strategy depends on the balance between competing evolutionary processes.

## Discussion

Eq (4) clearly reveals how high-dose chemotherapy gives rise to opposing evolutionary processes in the emergence of resistance. It shows how the balance between mutation and competition determines the optimal resistance management strategy [13, 19]. Increasing the drug concentration reduces mutational inputs into the system but it also unavoidably reduces the ecological control of any HLR pathogens that are present. These opposing forces typically generate an evolutionary hazard curve that is unimodal. Consequently, the worst approach is to treat with intermediate doses (Fig 1). The best approach is to administer either the largest tolerable dose or the smallest clinically effective dose (that is, the concentration at either end of the therapeutic window). Which of these is optimal depends on the relative positions of the hazard curve and the therapeutic window (Fig 1). Administering the highest tolerable dose can be a good strategy (Fig 1c and 1d) but it can also be less than optimal (Fig 1b) or even the worst thing to do (Fig 1a). Thus, nothing in evolutionary theory supports the contention that a ‘hit-hard’ strategy is a good rule of thumb for resistance management.

### Empirical evidence

Our framework makes a number of empirical predictions that are consistent with existing data. First, the resistance hazard will be maximized at intermediate drug concentrations. This is well-verified in numerous studies. In fact a unimodal relationship between resistance emergence and drug concentration (often called an ‘inverted-U’ in the literature) is arguably the single-most robust finding in all of the empirical literature [23–41].

Second, the position and shape of the hazard curve will vary widely among drugs and microbes, depending on how drug dose affects mutation rates and the strength of competition. Such wide variation is seen [23, 24, 28, 29, 35, 38, 39, 42, 43], presumably reflecting variation in the strength of the opposing processes highlighted by Eq (4).

Third, the relationship found between drug concentration and resistance evolution in any empirical study will depend on the range of concentrations explored. At the low end, increasing dose should increase resistance evolution; at the high end, increasing dose should decrease resistance evolution. Examples of both cases are readily seen, often even within the same study [15, 23–41, 44–50]. It is important to note that there are clear examples for which low-dose treatments can better prevent resistance emergence than high doses [15, 39, 42, 44–47, 49–51], despite an inherent focus in the literature on experimental exploration of high-dose chemotherapy. The theory presented here argues that uniformity is not expected and the bulk of the empirical literature is consistent with this prediction.

### Theory does not support using the MPC as a rule of thumb

An important and influential codification of Ehrlich’s ‘hit hard’ philosophy is the concept of the mutant selection window, and the idea that there exists a mutant prevention concentration (MPC) that best prevents resistance evolution [7–9]. The MPC is defined as ‘the lowest antibiotic concentration that prevents replication of the least susceptible single-step mutant’ (see p. S132 in ref. [8]). When drug concentrations are maintained above the MPC, ‘pathogens populations are forced to acquire two concurrent resistance mutations for replication under antimicrobial therapy’ (see p. 731 in ref. [52]). Below the MPC lies the ‘mutant selection window’, where single-step resistant mutants can replicate, thus increasing the probability that microbes with two or more resistance mutations will appear. Considerable effort has been put into estimating the MPC for a variety of drugs and microbes [4].

The relationship between these ideas and the theory presented here is best seen using the extension of Eq (4) that allows for strains with intermediate resistance. Appendix 2 in S1 Text shows that, in this case, Eq (4) remains unchanged except that its first term (the mutational component) is extended to account for all of the ways in which the HLR strain can arise by mutation through strains with intermediate resistance (see expression 2–3 in Appendix 2 of S1 Text). A focus on the MPC can therefore be viewed as a focus on trying to control only the mutational component of resistance emergence. And as the theory embodied by Eq (4) shows, doing so ignores the other evolutionary process of competitive release that is operating. The use of the MPC therefore cannot be supported by evolutionary theory as a general rule of thumb for resistance management.

If evolutionary theory does not support the use of MPC as a general approach then why does this nevertheless appear to work in some cases [34, 53]? The theory presented here provides some possible explanations. First, if HLR strains can appear only through mutation from strains with intermediate resistance, and if feasible dosing regimens can effectively kill all first step mutants, then such an approach must necessarily work since it reduces all mutational input to zero. But for most of the challenging resistance management situations in medicine, achieving this is presumably not possible. For example, if the MPC is not delivered to all pathogens in a population because of patient compliance, metabolic variation, spatial heterogeneity in concentration, etc, then the mutational input will not be zero. Also, if HLR strains can arise in ways that do not require mutating through strains with intermediate resistance (e.g., through lateral gene transfer; [54]) then again the mutational input will not be zero. In either case, one must then necessarily account for how the choice of dose affects the opposing evolutionary process of competitive release in order to minimize the emergence of resistance. S3 Fig illustrates this idea by presenting a numerical example in which the MPC is the worst choice of drug concentration for controlling HLR.

Second, the theory presented here suggests that the MPC *can* be the best way to contain resistance if this concentration happens to be the upper bound of the therapeutic window (although see S3 Fig for a counterexample). If, however, the MPC is less than the upper bound then even better evolution-proofing should be possible at either end of the therapeutic window. If the MPC is greater than the upper bound, as it is for example with most individual TB drugs [55] and levofloxacin against *S. aureus* [28], the MPC philosophy is that the drug should then be abandoned as monotherapy. But our framework suggests that before doing so, it might be worthwhile considering the lower bound of the therapeutic window. Researchers have tended not to examine the impact of the smallest clinically effective dose on resistance evolution, perhaps because of an inherent tendency to focus on high-dose chemotherapy. It would be informative to compare the effects of the MPC with concentrations from both ends of the therapeutic window on resistance emergence experimentally.

### Theory does not support using the highest tolerable dose as a rule of thumb

The MPC has yet to be estimated for many drug-microbe combinations [4] and it can be difficult to do so, especially in a clinically-relevant setting [52, 54]. Given the uncertainties involved, and the need to make clinical decisions ahead of the relevant research, some authors have suggested the working rule of thumb of administering the highest tolerable dose [3, 4]. Our analysis shows that evolutionary theory provides no reason to expect that this approach is best. By reducing or eliminating the only force which retards the emergence of any HLR strains that are present (i.e., competition), Eq (4) makes clear that a hit hard strategy can backfire, promoting the very resistance it is intended to contain.

### How to choose dose

If the relative positions of the HLR hazard curve and the therapeutic window are known, rational (evidence-based) choice of dose is possible. If the therapeutic window includes doses where the resistance hazard is zero, then those doses should be used. However, by definition, such situations are incapable of generating the HLR which causes a drug to be abandoned, and so these are not the situations that are most worrisome. If the hazard is non-zero at both ends of the therapeutic window, the bound associated with the lowest hazard should be used (Fig 1b and 1c). If nothing is known of the HLR hazard curve (as will often be the case), then there is no need to estimate the whole function. Our analysis suggests that the hazards need be estimated only at the bounds of the therapeutic window. These bounds are typically well known because they are needed to guide clinical practice. Estimating the resistance hazard experimentally can be done in vitro and in animal models but we note that since the solution falls at one end of the therapeutic window, they can also be done practically and ethically in patients. That will be an important arena for testing, not least because an important possibility is that, as conditions change, the optimal dose might change discontinuously from the lowest effective dose to the highest tolerable dose or vice versa. There is considerable scope to use mathematical and animal models to determine when that might be the case and to determine clinical predictors of when switches should be made.

### Managing resistance in non-targets

Our focus has been on the evolution of resistance in the pathogen population responsible for disease. Looking forward, an important empirical challenge is to consider the impact of drug dose on the broader microbiome. Resistance can also emerge in non-target micro-organisms in response to the clinical use of antimicrobials [45]. Resistance in those populations can increase the likelihood of resistance in future pathogen populations, either because of lateral gene transfer from commensals to pathogens, or when commensals become opportunistic pathogens [9, 56, 57]. For instance, aggressive drug treatment targeted at bacterial pneumonia in a rat model selected for resistance in gut fauna. Lower dose treatment of the targeted lung bacteria was just as clinically effective and better managed resistance emergence in the microbiota [51].

It is unclear just how important these off-target evolutionary pressures are for patient health, but if they are quantitatively important, this raises the interesting and challenging possibility that the real hazard curve should be that of the collective microbiome as a whole, weighted by the relative risk of resistance evolution in the components of the microbiome and the target pathogen. It will be challenging to determine that, but our focus on either end of the therapeutic window at least reduces the parameter space in need of exploration.

### Coda

Our analysis suggests that resistance management is best achieved by using a drug concentration from one edge of the therapeutic window. In practice, patients are likely treated more aggressively than the minimum therapeutic dose (to ensure no patients fail treatment) and less aggressively than the maximum tolerable dose (to ensure no patients suffer toxicity). This means that medical caution is always driving resistance evolution faster than it need go, particularly when the maximum hazard lies within the therapeutic window (Fig 1b and 1c). From the resistance management perspective, it is important to determine the level of caution that is clinically warranted rather than simply perceived.

For many years, physicians have been reluctant to shorten antimicrobial courses, using long courses on the grounds that it is better to be safe than sorry. It is now increasingly clear from randomized trials that short courses do just as well in many cases [58–60] and they can reduce the risk of resistance emergence [58, 61, 62]. We suggest that analogous experiments looking at the evolutionary outcomes of lowest clinically useful doses should be the next step. Such experiments in plants have already shown unambiguously that low dose fungicide treatment best prevents the spread of resistant fungal pathogens [63]. How generally true this is for other pathogens, or pathogens of other hosts, remains to be seen. We also note that our arguments about the evolutionary merits of considering the lowest clinically useful doses have potential relevance in the evolution of resistance to cancer chemotherapy as well [64].

## Supporting Information

S1 Text Appendix 1—Derivation of Eq 4; Appendix 2—Extensions involving intermediate strains and horizontal gene transfer; Appendix 3—A Model of acute immune-mediated infections; Appendix 4—Other results for the model of acute immune-mediated infections; Appendix 5—A Model of chronic infection based on resource competition; Appendix 6—Generalizing the pharmacokinetics.
(PDF)

S1 Fig Dynamics of model in the absence of resistance. (a) The dose-response curve *r*(*c*) = 0.6(1−tanh(15(*c*−0.3))) as well as the therapeutic window in green. (b), (c) and (d) show wild type pathogen density (blue) and immune molecule density (black) during infection for 1000 representative realizations of a stochastic implementation of the model. (b) no treatment, (c) treatment at the smallest effective dose *c*_*L*_, (d) treatment at the maximum tolerable dose *c*_*U*_. Parameter values are *P*(0) = 10, *I*(0) = 2, *α* = 0.05, *δ* = 0.05, *κ* = 0.075, *μ* = 0, and *γ* = 0.01.
(TIF)

S2 Fig The effect of different levels of standing variation for resistance in the initial infection. Simulation is identical to that for Fig 3a except for the initial conditions. The dose-response curves for the wild type in blue (*r*(*c*) = 0.6(1−tanh(15(*c*−0.3)))) and the resistant strain in red (*r*_*m*_(*c*) = 0.59(1−tanh(15(*c*−0.6)))) as well as the therapeutic window in green. Red dots indicate the probability of resistance emergence, and for three different initial conditions. Probability of resistance emergence is defined as the fraction of 5000 simulations for which resistance reached a density of at least 100 (and thus caused disease). Top set of dots have *P*(0) = 5, *P*_*m*_(0) = 5; middle set of dots have *P*(0) = 7, *P*_*m*_(0) = 3; bottom set of dots have *P*(0) = 10, *P*_*m*_(0) = 0. Other parameter values are *I*(0) = 2, *α* = 0.05, *δ* = 0.05, *κ* = 0.075, *μ* = 10^−2^, and *γ* = 0.01.
(TIF)

S3 Fig Simulation results when there is a strain with intermediate resistance. (a) The dose-response curves for the wild type in blue (*r*(*c*) = 0.6(1−tanh(15(*c*−0.3)))), the intermediate strain in yellow (*r*_*m*2_(*c*) = 0.595(1−tanh(15(*c*−0.45)))), and the HLR strain in red (*r*_*m*2_(*c*) = 0.59(1−tanh(15(*c*−0.6)))) as well as the therapeutic window in green. Dots indicate the probability of emergence for the intermediate strain (yellow) and the HLR strain (red). Probability of emergence is defined as the fraction of 5000 simulations for which the strain reached a density of at least 100. (b) and (c) wild type density (blue), intermediate strain density (yellow), HLR strain density (red), and immune molecule density (black) during infection for 1000 representative realizations of a stochastic implementation of the model. (b) treatment at the smallest effective dose *c*_*L*_, (c) treatment at the maximum tolerable dose *c*_*U*_. Parameter values are *P*(0) = 10, *P*_*m*1_(0) = 0, *P*_*m*2_(0) = 0, *I*(0) = 2, *α* = 0.05, *δ* = 0.05, *κ* = 0.075, *μ* = 10^−2^, *μ*_1_ = 10^−2^, and *γ* = *γ*_*m*1_ = *γ*_*m*2_ = 0.01.
(TIF)

S4 Fig The effect of drug concentration on resistance emergence and treatment failure. (a) The dose-response curves for the wild type in blue (*r*(*c*) = 0.6(1−tanh(15(*c*−0.3)))) and the resistant strain in red (*r*_*m*_(*c*) = 0.59(1−tanh(15(*c*−0.6)))) as well as the therapeutic window in green. Dots indicate the probability of resistance emergence. Probability of resistance emergence is defined as the fraction of 5000 simulations for which resistance reached a density of at least 100 (and thus caused disease). Parameter values are *P*(0) = 10, *I*(0) = 2, *α* = 0.05, *δ* = 0.05, *κ* = 0.075, *μ* = 10^−2^, and *γ* = 0.01. Bar graphs: the probability that a resistant strain appears by mutation is indicated by the left-hand grey bars for each drug concentration (the right-hand grey bar is the probability that a resistant strain does not appear). The probability of treatment failure for a specific drug dose is the sum of the red bars for that dose. (b) Same as panel (a) but with mutation rate decreased to *μ* = 10^−3^.
(TIF)

S5 Fig Dynamics of chronic infection in the absence of resistance. (a) The dose-response curve *r*(*c*) = 0.00255(1−tanh(15(*c*−0.3))) as well as the therapeutic window in green. (b), (c) and (d) show wild type pathogen density (blue) and resource density (black) during infection for 20 representative realizations of a stochastic implementation of the model. (b) no treatment, (c) treatment at the smallest effective dose *c*_*L*_, (d) treatment at the maximum tolerable dose *c*_*U*_. Parameter values are *P*(0) = 2, *R*(0) = 2000, *θ* = 200, *δ* = 0.1, *d* = 2, and *μ* = 0.
(TIF)

S6 Fig Example where conventional strategy of high-dose chemotherapy best prevents the emergence of resistance. (a) The dose-response curves for the wild type in blue (*r*(*c*) = 0.00255(1−tanh(15(*c*−0.3)))) and the resistant strain in red (*r*_*m*_(*c*) = 0.0025(1−tanh(15(*c*−0.45)))) as well as the therapeutic window in green. Red dots indicate the probability of resistance emergence. Probability of resistance emergence is defined as the fraction of 1000 simulations for which resistance reached a density of at least 300 (and thus caused disease). (b) and (c) wild type density (blue), resistant density (red), and resource density (black) during infection for 20 representative realizations of a stochastic implementation of the model. (b) treatment at the smallest effective dose *c*_*L*_, (c) treatment at the maximum tolerable dose *c*_*U*_. Parameter values: *P*(0) = 2, *P*_*m*_(0) = 0, *R*(0) = 2000, *θ* = 200, *δ* = 0.1, *d* = 2, *d*_*m*_ = 2.7, and *μ* = 10^−2^.
(TIF)

S7 Fig Example where a low-dose strategy best prevents the emergence of resistance. (a) The dose-response curves for the wild type in blue (*r*(*c*) = 0.00255(1−tanh(15(*c*−0.3)))) and the resistant strain in red (*r*_*m*_(*c*) = 0.0025(1−tanh(15(*c*−0.6)))) as well as the therapeutic window in green. Red dots indicate the probability of resistance emergence. Probability of resistance emergence is defined as the fraction of 1000 simulations for which resistance reached a density of at least 300 (and thus caused disease). (b) and (c) wild type density (blue), resistant density (red), and resource density (black) during infection for 20 representative realizations of a stochastic implementation of the model. (b) treatment at the smallest effective dose *c*_*L*_, (c) treatment at the maximum tolerable dose *c*_*U*_. Parameter values: *P*(0) = 2, *P*_*m*_(0) = 0, *R*(0) = 2000, *θ* = 200, *δ* = 0.1, *d* = 2, *d*_*m*_ = 2.7, and *μ* = 10^−2^.
(TIF)
