# Supplementary material for: Does High-Dose Antimicrobial Chemotherapy Prevent the Evolution of Resistance?
Source: PLoS Comput Biol. 2016 Jan 28;12(1):e1004689. doi: 10.1371/journal.pcbi.1004689 (PMC4731197; doi:10.1371/journal.pcbi.1004689)
Supplement: S1 Text — Appendix 1—Derivation of Eq 4; Appendix 2—Extensions involving intermediate strains and horizontal gene transfer; Appendix 3—A Model of acute immune-mediated infections; Appendix 4—Other results for the model of acute immune-mediated infections; Appendix 5—A Model of chronic infection based on resource competition; Appendix 6—Generalizing the pharmacokinetics. (PDF) [file pcbi.1004689.s001.pdf]

Supporting Information

When does high-dose antimicrobial chemotherapy prevent the evolution of resistance?

Day, T. and A.F. Read

October 25, 2015

Appendix 1 - Derivation of Equation 4

Appendix 2 - Extensions involving intermediate strains and horizontal gene transfer

Appendix 3 - A Model of acute immune-mediated infections

Figure S1

Figure S2

Figure S3

Appendix 4 - Other results for the model of acute immune-mediated infections

Figure S4

Appendix 5 - A model of chronic infection based on resource competition

Figure S5

Figure S6

Figure S7

Appendix 6 - Generalizing the pharmacokinetics

Appendix 1 - Derivation of Equation 4

In the absence of treatment we model the within-host dynamics using a system of differential equations

$$\frac{dP}{dt} = F(P, X) \quad (1\text{-}1a)$$
$$\frac{dX}{dt} = G(P, X) \quad (1\text{-}1b)$$

where P is the density of the wild type and X is a vector of variables describing the within-host state (e.g., RBC count, densities of different immune molecules, etc). The initial conditions are $P(0) = P_0$ $X(0) = X_0$. At some point, t^*, drug treatment is introduced. Using lower case letters to denote the dynamics in the presence of treatment, we then have

$$\frac{dp}{dt} = f(p, x; c) \quad (1\text{-}2a)$$
$$\frac{dx}{dt} = g(p, x; c) \quad (1\text{-}2b)$$

with initial conditions $p(0; c) = P(t^*)$ and $x(0; c) = X(t^*)$, and where c is the dosage. For simplicity, here we assume that a constant drug concentration is maintained over the course of the infection. Appendix 6 considers the pharmacokinetics of discrete drug dosing. The notation $p(t; c)$ and $x(t; c)$ reflects the fact that the dynamics of the wild type and the host state will depend on dosage. For example, if the dosage is very high p will be driven to zero very quickly.

As the drug removes the wild type pathogen, resistant mutations will continue to arise from the wild type population stochastically. For example, if mutations are produced only during replication of the wild type, then the rate of mutation will have the form $\mu r(c)p(t;c)$ where μ is the mutation rate and $r(c)$ is the replication rate of the wild type pathogen (which depends on drug dosage c). With this form of mutation, if we could administer the drug at concentrations above the MIC at the very onset of infection, then resistance evolution through *de novo* mutation would not occur. In reality symptoms and therefore drug treatment typically do not occur until later in the infection, meaning that some resistant strains might already be present at low frequency at the onset of treatment. There are also other plausible forms for the mutation rate as well, and therefore we simply specify this rate by some general function $\lambda[p(t;c),c]$.

Whenever a resistant strain appears it is subject to stochastic loss. We define π as the probability of avoiding loss (which we refer to as 'escape'). To simplify the present analysis, we use a separation of timescales argument and assume that the fate of each mutant is determined quickly (essentially instantaneously) relative to the dynamics of the wild type and host state (we relax this assumption in all numerical examples). Thus, π for any mutant will depend on the host state at the time of its appearance, $x(t;c)$, and it will therefore depend indirectly on c. Note that π will also depend directly on c, however, because drug dosage might directly suppress resistant strains as well if the dose is high enough. Therefore we use the notation $\pi[x(t;c),c]$, and assume that π is an increasing function of x and a decreasing function of c.

With the above assumptions the host can be viewed as being in one of two possible states at any point in time during the infection: (i) resistance has emerged (i.e., a resistant strain has appeared and escaped), or (ii) resistance has not emerged. We model emergence as an inhomogeneous birth process, and define $q(t)$ as the probability that resistance has emerged by time t. A conditioning argument gives

$$q(t + \Delta t) = q(t) + (1 - q(t))\lambda \Delta t \pi + o(\Delta t) \tag{1-3}$$

where $\lambda \Delta t$ is the probability that a mutant arises in time Δt, and π is the probability that such a mutant escapes. Re-arranging and taking the limit $\Delta t \to 0$ we obtain

$$\frac{dq}{dt} = (1 - q(t))\lambda \pi \tag{1-4}$$

with initial condition $q(0) = q_0$. Note that q_0 is the probability that emergence occurs as a result of resistant mutants being present at the start of treatment. Again employing a separation of timescales argument, if there are n mutant individuals present at this time, then $q_0 = 1 - (1 - \pi[x(0; c), c])^n$.

The solution to the above differential equation is

$$q(t) = 1 - (1 - \pi[x(0; c), c])^n \exp\left(-\int_0^t \lambda \pi \mathrm{d}s\right). \tag{1-5}$$

If a is the time at which treatment is stopped, and Q is the probability of emergence occurring at some point during treatment, then $Q = q(a)$. If we further define $S = -n \ln(1 - \pi[x(0; c), c])$ then we can write Q as

$$Q = 1 - \exp(-D - S) \tag{1-6}$$

where $D = \int_0^a \lambda \pi \mathrm{d}s$. We refer to D as the *de novo* hazard and S as the standing hazard. D is the contribution to escape that is made up of mutant microbes that arise during the course of treatment. S is the contribution to escape that is made up of mutant microbes

already presents at the start of treatment.

Given the expression for Q, all else equal, resistance management would seek the treatment strategy, c that makes Q as small as possible. Since Q is a monotonic function of $D+S$, we can simplify matters by focusing on these hazards instead. Thus we define

$$H = \int_0^a \lambda\pi \mathrm{d}s + S \tag{1-7}$$

which is the 'total hazard' during treatment. Equation (4) is then obtained by differentiating the the total hazard H with respect to c.

All of the simulation results presented in the main text and in the supporting information show that hazard $H(c)$ is a unimodal function of dose. Indeed we have not found a case where the hazard has a more complicated shape, and the extensive body of empirical results discussed in the main text show that a unimodal relationship is the norm as well. Nevertheless, in principle there is no obvious reason why the hazard couldn't have a multimodal shape. Even in such cases, however, once noise is introduced in the form of physiological variation across treated individuals, this will tend to result in an overall unimodal relationship.

To see why, suppose that a dose c is administered but physiological variation results in the realized dose taking on a different value α. We define $p(\alpha; c)$ to be the probability density that α is the realized dose in a randomly chosen patient when the administered dose is c. If we then compute the average hazard over all individuals treated with dose c we get $\tilde{H}(c) = \int_0^a H(\alpha)p(\alpha; c)d\alpha$. Now if we expand $H(\alpha)$ in a Taylor series around c we get

$$\begin{aligned}\tilde{H}(c) &= \int_0^a \{H(c) + H'(c)(\alpha - c) + \frac{H''(c)}{2}(\alpha - c)^2 + \cdots\} p(\alpha; c) d\alpha \\ &= H(c) + H'(c)\int_0^a (\alpha - c) p(\alpha; c) d\alpha + \frac{H''(c)}{2}\int_0^a (\alpha - c)^2 p(\alpha; c) d\alpha + \cdots\end{aligned}$$

Finally, if the variation is small and unbiased (i.e., the mean value of α for an administered dose of c is c) then this can be approximated as

$$\tilde{H}(c) \approx H(c) + \frac{H''(c)}{2}\sigma_c^2 \tag{1-8}$$

where σ_c^2 is the variance in the realized dose for an administered dose of c. From (1-8) we can see that physiological variation will tend to increase the value of the average hazard $\tilde{H}(c)$ compared to the function $H(c)$ for values of c where $H(c)$ is concave up (i.e., $H''(c) > 0$) and it will tend to decrease the value of the average hazard $\tilde{H}(c)$ compared to the function $H(c)$ for values of c where $H(c)$ is concave down (i.e., $H''(c) < 0$). This means that $\tilde{H}$ will tend to be a 'smoothed' version of $H(c)$, where the dips are filled in and the peaks are lowered.

Appendix 2 - Extensions involving intermediate strains and horizontal gene transfer

The results of the main text (which are derived in Appendix 1) are based on the assumption that a single mutational event can give rise to high-level resistance. In some situations several mutational events might be required. These so-called 'stepping stone mutations' towards high-level resistance might themselves confer an intermediate level of resistance. One of the arguments in favour of aggressive chemotherapy has been to prevent the persistence of these stepping stone strains, and thereby better prevent the emergence of high-level resistance [1–8]. Here we incorporate such stepping stone mutations into the theory, again placing primary attention on the emergence of high-level resistance.

As in Appendix 1, in the absence of treatment we model the within-host dynamics using a system of differential equations

$$\frac{dP}{dt} = F(P, X) \tag{2-1a}$$
$$\frac{dX}{dt} = G(P, X) \tag{2-1b}$$

but now P is also a vector containing the density of the wild type and all potential intermediate mutants. All intermediate strains are assumed to bear some metabolic or replicative cost as well, meaning that they are unable to increase in density in the presence of the wild type. Mechanistically again this is because the wild type has suppressed the host state, X, below the minimum value required for a net positive growth by any intermediate strain. Thus, in the absence of treatment we expect most of these mutants to have negligible density. Once treatment is introduced we have

$$\frac{dp}{dt} = f(p, x; c) \tag{2-2a}$$

$$\frac{dx}{dt} = g(p, x; c) \tag{2-2b}$$

where again p is now a vector. As before we have initial conditions $p(0; c) = P(t^*)$ and $x(0; c) = X(t^*)$, and where c is the dosage. Now, however, different choices of c will generate different distributions of strain types $p(t; c)$ during the infection. Furthermore, each type will give rise to the high-level resistance strain with its own rate. Therefore, the function specifying the rate of mutation to the HLR strain $\lambda[p(t; c), c]$ is a function of the vector variable $p(t; c)$.

The calculations in Appendix 1 can again be followed. We obtain an equation identical to equation (4) except that the first term is replaced by

$$\int_0^a \pi \left(\nabla_p \lambda \cdot p_c + \frac{\partial \lambda}{\partial c} \right) \mathrm{d}s \tag{2-3}$$

where subscripts denote differentiation with respect to that variable. The difference is that $(\partial\lambda/\partial p)(\partial p/\partial c)$ in equation (4) is replaced with $\nabla_p \lambda \cdot p_c$. The quantity p_c is a vector whose components are the changes in the density of each intermediate strain arising from an increased dosage. The quantity $\nabla_p \lambda$ is the gradient of the mutation rate with respect to a change in the density of each intermediate strain. The integral of the dot product of the two, $\nabla_p \lambda \cdot p_c$, is therefore the overall change in mutation towards the HLR strain during treatment. Whereas the first term of equation (4) is expected to be negative, expression (2-3) can be negative or positive depending on how different doses affect the distribution of intermediate mutants during the infection (i.e., the elements of p_c) and the rate at which each type of intermediate mutant gives rise to the strain with high level resistance (i.e., the

elements of $\nabla_p \lambda$). Either way, however, this does not alter the salient conclusion that the optimal resistance management dose will depend on the details.

In an analogous fashion we might also alter the derivation in Appendix 1 to account for the possibility that some microbes acquire high-level resistance via horizontal gene transfer from other, potentially commensal, microbes. To do so we would simply need to alter the way in which λ is modelled. In particular, it might then be a function of the densities of commensal microbes as well, who themselves could be affected by drug dosage. Thus, once treatment has begun, we might have a system of equations of the form

$$\frac{dp}{dt} = f(p, x, y; c) \tag{2-4a}$$

$$\frac{dx}{dt} = g(p, x, y; c) \tag{2-4b}$$

$$\frac{dy}{dt} = h(p, x, y; c) \tag{2-4c}$$

where y is a vector of commensal microbe densities. We might then model λ as $\lambda[p(t; c), y(t; c)]$. Again, calculations analogous to those of Appendix 1 can be followed to obtain an appropriate expression for the resistance hazard. As with the above examples, there will again be a tradeoff between components of this expression as a function of drug dosage.

Appendix 3 - A Model of acute immune-mediated infections

The dynamics of the mutant and wild type in the absence of treatment are modeled as

$$\frac{dP}{dt} = [r(0)(1-\mu)-\gamma]P - \kappa P I \quad (3\text{-}1)$$

$$\frac{dP_m}{dt} = [r_m(0)-\gamma_m]P_m - \kappa P_m I + r(0)\mu P \quad (3\text{-}2)$$

$$\frac{dI}{dt} = \alpha(P+P_m) - \delta I. \quad (3\text{-}3)$$

where $r(\cdot)$ and $r_m(\cdot)$ are the growth rates of the wild type and mutant as a function of drug concentration, μ is the mutation probability from wild type to resistant, and γ and γ_m are the natural death rates of each. We assume a cost of resistance in the absence of treatment, meaning that $r(1-\mu)-\gamma > r_m - \gamma_m$ The immune response, I, grows in proportion to the density of the pathogen population and decays at a constant per capita rate δ. Immune molecules kill the pathogen according to a law of mass action with parameter κ for both the wild type and the resistant strain (i.e., immunity is completely cross-reactive). This is a simple deterministic model for an immune-controlled infection.

When the mutation rate is zero ($\mu = 0$) and the pathogen can increase when rare, the model displays damped oscillations towards an equilibrium with the wild type present ($\hat{P} = (r-\gamma)\delta/\alpha\kappa$), the mutant extinct ($\hat{P_m} = 0$), and the immune system at a nonzero level ($\hat{I} = (r-\gamma)/\kappa$). For many choices of parameter values (including those that we focus on here) the first trough in pathogen density is very low, and therefore once we introduce stochasticity the entire pathogen population typically goes extinct at this stage, at which point the immune molecules then decay to zero. It is in this way that we model an

immune-controlled infection.

Under treatment the dynamics are the same as above but where $r(\cdot)$ and $r_m(\cdot)$ are then evaluated at some nonzero drug concentration. Throughout we assume that the dose-response functions $r(\cdot)$ and $r_m(\cdot)$ are given by the function $b_1(1 - \tanh(b_2(c - b_3)))$ for some constants b_1, b_2, and b_3. The model used to explore the emergence of resistance employs a stochastic implementation of the above equations using the Gillespie algorithm.

Figure S1 presents output for several runs of the model using three different drug concentrations. In all cases we have set the mutation rate to zero (no resistant strains arise). In the absence of treatment an infection typically results in a single-peak of wild type pathogen before the infection is cleared. To model realistic disease scenarios we (arbitrarily) suppose that infected individuals become symptomatic only once the pathogen density exceeds a threshold of 100 and treatment is used only once an infection is symptomatic. For the parameter values chosen in this example, 99% of untreated infections are symptomatic (Figure S1a,b). We further suppose (again arbitrarily) that a pathogen load greater than 200 results in substantial morbidity and/or mortality. With these assumptions we can then proceed to define the therapeutic window. The upper limit c_U is arbitrary in the model and so we set $c_U = 0.5$. The lower limit c_L is the smallest dose that prevents significant morbidity and/or mortality. Therefore it is the smallest dose that, in the absence of resistance emergence, keeps pathogen load below 200. Figure S1c shows that, for the parameter values used, $c_L \approx 0.3$. Notice from Figure S1a that a dose of $c = 0.3$ does not fully suppress growth as measured *in vitro* but it nevertheless controls the infection *in vivo* because the immune response also contributes to reducing the pathogen load.

Figure S1. Dynamics in the absence of resistance. (a) The dose-response curve $r(c) = 0.6(1 - \tanh(15(c - 0.3)))$ as well as the therapeutic window in green. (b), (c) and (d) show wild type pathogen density (blue) and immune molecule density (black) during infection for 1000 representative realizations of a stochastic implementation of the model. (b) no treatment, (c) treatment at the smallest effective dose c_L, (d) treatment at the maximum tolerable dose c_U. Parameter values are $P(0) = 10$, $I(0) = 2$, $\alpha = 0.05$, $\delta = 0.05$, $\kappa = 0.075$, $\mu = 0$, and $\gamma = 0.01$.

For simulations in which the mutation rate to resistance is non-zero we quantify the emergence of resistance in the following way. For each simulation run we record the maximum density of the resistant strain before the infection is ultimately cleared. Runs in which this density reaches a level high enough to cause symptoms (a density of 100 in this case) are deemed to be infections in which resistance has emerged. The probability of resistance emergence is quantified as the fraction of runs in which this threshold level is reached. In Figure 4 of the text we also consider the consequences of using other threshold densities to define emergence.

The simulation results of the main text assume that all resistant strains arise *de novo* in a infection but in some cases we might expect resistant strains to already be present at the start of infection. The general theory presented in the main text reveals that again we should not expect any simple generalities. For example, one might expect that when the initial infection already contains many resistant microbes the relevance of *de novo* mutation might be diminished and so a lower dose might be optimal for managing resistance. Although this is sometimes the case (Day, unpubl. results) the opposite is possible as well.

As an example, Figure S2 presents results for the probability of emergence as a function of dose, for three different levels of resistance frequency in the initial infection. As the

frequency of resistance in the initial infection increases, the optimal concentration changes from a low dose to a high dose. The reason is that, if resistance is already very common early in the infection, then the competitive release that occurs from removing the wild type is greatly diminished since the resistant strain will have already managed to gain a foothold before the wildtype numbers increase significantly. Put another way, the benefits of low dose therapy have decreased because the magnitude of competitive release (the blue terms in equation (4) of the main text) has decreased. Experimental results have verified this prediction; namely, that drug resistant pathogens can reach appreciable within-host densities in the absence of treatment if the initial infection contains a substantial number of these [9].

Figure S2. The effect of different levels of standing variation for resistance in the initial infection. Simulation is identical to that for Figure 3a except for the initial conditions. The dose-response curves for the wild type in blue ($r(c) = 0.6(1 - \tanh(15(c-0.3)))$) and the resistant strain in red ($r_m(c) = 0.59(1-\tanh(15(c-0.6)))$) as well as the therapeutic window in green. Red dots indicate the probability of resistance emergence, and for three different initial conditions. Probability of resistance emergence is defined as the fraction of 5000 simulations for which resistance reached a density of at least 100 (and thus caused disease). Top set of dots have $P(0) = 5$, $P_m(0) = 5$; middle set of dots have $P(0) = 7$, $P_m(0) = 3$; bottom set of dots have $P(0) = 10$, $P_m(0) = 0$. Other parameter values are $I(0) = 2$, $\alpha = 0.05$, $\delta = 0.05$, $\kappa = 0.075$, $\mu = 10^{-2}$, and $\gamma = 0.01$.

A common suggestion is that, when strains with intermediate levels of resistance are possible, aggressive chemotherapy is then optimal because anything less will allow these intermediate strains to persist and thereby give rise to HLR through mutation. We therefore conducted simulations to explore this idea. We note, however, that again the general theoretical results of Appendix 2 reveal that no generalities should be expected and

our simulations bear this out. For example, we extended equations for the within-host dynamics to allow for a strain with intermediate resistance by using the following equations:

$$\frac{dP}{dt} = [r(c)(1-\mu)-\gamma]P - \kappa PI \tag{3-4}$$

$$\frac{dP_{m1}}{dt} = [r_{m1}(c)-\gamma_{m1}]P_{m1} - \kappa P_{m1}I + r(c)\mu P \tag{3-5}$$

$$\frac{dP_{m2}}{dt} = [r_{m2}(c)-\gamma_{m2}]P_{m2} - \kappa P_{m2}I + r_{m1}(c)\mu_1 P_{m1} \tag{3-6}$$

$$\frac{dI}{dt} = \alpha(P + P_{m1} + P_{m2}) - \delta I. \tag{3-7}$$

where P_{m1} is the density of the mutant strain with intermediate resistance and P_{m2} is the strain with HLR. Also, $r(\cdot)$, $r_{m1}(\cdot)$, and $r_{m2}(\cdot)$ are the growth rates of the wild type and the two mutant types as a function of drug concentration, μ is the mutation probability from wild type to the intermediate strain, μ_1 is the mutation rate from the intermediate strain to HLR, and the γ's are the natural death rates of each. Again the immune response, I, grows in proportion to the density of the pathogen population and decays at a constant per capita rate δ.

Again the simulation was conducted with a stochastic implementation of the above model using the Gillespie algorithm. While the presence of intermediate strains does alter the relative balance of factors affecting resistance emergence, this balance can still move in either direction.

As an example, Figure S3 presents simulation results in which low-dose treatment yields the lowest probability of HLR emergence. Note, however, that high-dose treatment controls the emergence of the intermediate strain the best.

Figure S3. Simulation results when there is a strain with intermediate resistance. (a) The dose-response curves for the wild type in blue ($r(c) = 0.6(1-\tanh(15(c-0.3)))$), the intermediate strain in yellow ($r_{m2}(c) = 0.595(1 - \tanh(15(c - 0.45)))$), and the HLR strain in red ($r_{m2}(c) = 0.59(1 - \tanh(15(c - 0.6)))$) as well as the therapeutic window in green. Dots indicate the probability of emergence for the intermediate strain (yellow) and the HLR strain (red). Probability of emergence is defined as the fraction of 5000 simulations for which the strain reached a density of at least 100. (b) and (c) wild type density (blue), intermediate strain density (yellow), HLR strain density (red), and immune molecule density (black) during infection for 1000 representative realizations of a stochastic implementation of the model. (b) treatment at the smallest effective dose c_L, (c) treatment at the maximum tolerable dose c_U. Parameter values are $P(0) = 10$, $P_{m1}(0) = 0$, $P_{m2}(0) = 0$, $I(0) = 2$, $\alpha = 0.05$, $\delta = 0.05$, $\kappa = 0.075$, $\mu = 10^{-2}$, $\mu_1 = 10^{-2}$, and $\gamma = \gamma_{m1} = \gamma_{m2} = 0.01$.

The results of Figure S3 can also be interpreted within the context of the mutant selection window hypothesis and the mutant prevention concentration or MPC. The MPC is the drug concentration that prevents the emergence of all single-step resistant mutants. Figure S3 we can see that the emergence of the intermediate, single step, mutant strain is prevented by using the maximum tolerable dose. Nevertheless, even though the HLR strain can arise only by mutation from this intermediate strain, it is the lowest effective dose that best controls the emergence of HLR. The reason for this is that it is not possible to achieve the MPC early enough in the infection to prevent all mutational input from occurring because treatment starts only once symptoms appear. For the specific case illustrated in Figure S3 the possibility of HLR arising is then enough to tip the balance so that the lower edge of the therapeutic window is the best strategy for controlling HLR.

Appendix 4 - Other results for the model of acute immune-mediated infections

In the main text we focus on the emergence of the resistant strain but in many clinical studies researchers focus instead on successful treatment. For example, one common approach is to quantify the probability of treatment failure as a function of drug dose (or some proxy thereof). Such studies cannot provide information about resistance evolution *per se* but they nevertheless might involve a component of resistance evolution if this is one of the potential reasons for treatment failure.

We can explore a similar idea in the context of the model in the main text. Suppose we measure clinical success as the complete eradication of infection by day 20. In the simulations some individuals then display treatment failure because, through the stochasticity of individual infection dynamics, they fail to clear the infection by this time. Figure S4a presents the probability of treatment failure, measured by the fraction of the simulations for which the infection (wild type or resistant) was still present on day 20 for the model underlying Figure 3. Failure occurs under both treatment scenarios but it happens more frequently for the high dose treatment (compare red portion of bar graphs in Figure S4a). There is an important structure to these failures, however, that can be better appreciated by calculating the probability of failure by conditioning on whether or not a resistant mutation ever appeared during treatment; i.e.,

$$P(F) = P(F|M)P(M) + P(F|M^c)P(M^c) \tag{4-1}$$

where $P(F)$ is the probability of failure, $P(M)$ is the probability of a resistant mutation appearing during treatment ($P(M^c)$ is the probability that this doesn't occur), and $P(F|M)$ is the probability of failure given a resistant mutation appears (with $P(F|M^c)$ the

probability of failure given a resistant mutation does not appear). The bar graphs in Figure S4a show again that a high dose better controls the appearance of resistant mutations (i.e., $P(M)$ is lower for the high dose treatment), but if a resistant mutation does occur, then a high dose results in a greater likelihood of treatment failure (i.e., $P(F|M)$ is higher for the high dose treatment - note that this quantity can be interpreted graphically as the ratio of the red to grey bars). And in this case the latter effect overwhelms the former, making the probability of treatment failure $P(F)$ greater overall for the high dose treatment.

Figure S4. The effect of drug concentration on resistance emergence and treatment failure. (a) The dose-response curves for the wild type in blue ($r(c) = 0.6(1 - \tanh(15(c - 0.3)))$) and the resistant strain in red ($r_m(c) = 0.59(1 - \tanh(15(c - 0.6)))$) as well as the therapeutic window in green. Dots indicate the probability of resistance emergence. Probability of resistance emergence is defined as the fraction of 5000 simulations for which resistance reached a density of at least 100 (and thus caused disease). Parameter values are $P(0) = 10$, $I(0) = 2$, $\alpha = 0.05$, $\delta = 0.05$, $\kappa = 0.075$, $\mu = 10^{-2}$, and $\gamma = 0.01$. Bar graphs: the probability that a resistant strain appears by mutation is indicated by the left-hand grey bars for each drug concentration (the right-hand grey bar is the probability that a resistant strain does not appear). The probability of treatment failure for a specific drug dose is the sum of the red bars for that dose. (b) Same as panel (a) but with mutation rate decreased to $\mu = 10^{-3}$.

It is not difficult to obtain diametrically opposite results, however, with a small change in parameter values. Figure S4b show analogous results for the very same simulation, but where the probability of mutation is an order of magnitude lower. In this case we see that, even though a high dose results in a greater probability of failure if a resistant mutation appears, the effect is diminished such that, overall, the high dose results in a lower overall probability of failure. Notice also though that, even though a high dose results in a lower likelihood of treatment failure, it nevertheless still results in a higher probability of

resistance emergence during treatment. The former is measured only by whether or not the infection still persists on day 20 whereas the latter is measured by whether or not a large outbreak of resistance occurs at some point during treatment. This provides an example illustrating the general idea that treatment failure cannot be taken as a proxy for resistance emergence.

Appendix 5 - A Model of chronic infection based on resource competition

In this appendix we present some additional simulation results for a model of chronic infection. We assume that pathogen strains interact through competition for a common resource (e.g., red blood cells) and that the immune response is negligible. This latter assumption might apply for immuno-compromised individuals but our primary reason for making this assumption is to demonstrate that the conclusions of the main text do not depend on the microbes interacting primarily through a shared immune response. In particular, we will show that again a seemingly small change in parameter values alters the outcome from one where the largest tolerable dose is optimal to one where the smallest effective dose is optimal.

The equations for the resource, the wild type, and the resistant mutant are

$$\frac{dR}{dt} = \theta - \delta R - r(c)PR - r_m(c)P_mR \tag{5-1}$$

$$\frac{dP}{dt} = r(c)(1-\mu)PR - dP \tag{5-2}$$

$$\frac{dP_m}{dt} = r_m(c)P_mR - d_mP_m + r(c)PR\mu \tag{5-3}$$

where R is the resource concentration, $r(\cdot)$ and $r_m(\cdot)$ are the dose-response functions of the wild type and mutant for a drug concentration c, and the per capita replication rate of each type is governed by a type 1 functional response (i.e., $r(c)R$ and $r_m(c)R$ respectively). The constant θ is the rate at which resources enter the system, δ is the per capita rate at which resources are lost through decay, μ is the mutation probability from wild type to resistant, and d and d_m are the natural death rates of each. The model used to explore the

emergence of resistance employs a stochastic implementation of the above equations using the Gillespie algorithm.

When the mutation rate is zero ($\mu = 0$) and the pathogen can increase when rare, the model displays damped oscillations towards an equilibrium with the wild type present ($\hat{P} = (\theta r - \delta d)/(dr)$), the mutant extinct ($\hat{P_m} = 0$), and the resource at a nonzero level ($\hat{R} = d/r$). Occasionally the stochastic version of the model results in the pathogen population going extinct by chance but for the parameter values explored here most simulation runs result in a chronic infection.

Figure S5 presents output for several runs of the model using three different drug concentrations. In all cases the mutation rate is set to zero (no resistant strains arise). In the absence of treatment an infection typically results in a large peak of wild type pathogen and then the density stabilizes at a low, chronic, level. To model realistic disease scenarios we (arbitrarily) suppose that infected individuals become symptomatic only once the pathogen density exceeds a threshold of 300 and treatment is used only once an infection is symptomatic. We further suppose (again arbitrarily) that a pathogen load greater than 700 results in substantial morbidity and/or mortality. With these assumptions we can then proceed to define the therapeutic window. The upper limit c_U is arbitrary in the model and so we set $c_U = 0.5$. The lower limit c_L is the smallest dose that prevents significant morbidity and/or mortality. Therefore it is the smallest dose that, in the absence of resistance emergence, keeps pathogen load below 700. Figure S5c shows that, for the parameter values used, $c_L \approx 0.3$.

Figure S5. Dynamics of chronic infection in the absence of resistance. (a) The dose-response curve $r(c) = 0.00255(1 - \tanh(15(c - 0.3)))$ as well as the therapeutic window in green. (b), (c) and (d) show wild type pathogen density (blue) and resource density (black) during infection for 20 representative realizations of a stochastic implementation of the model. (b) no treatment, (c) treatment at the smallest effective dose c_L, (d) treatment at the maximum tolerable dose c_U. Parameter values are $P(0) = 2$, $R(0) = 2000$, $\theta = 200$, $\delta = 0.1$, $d = 2$, and $\mu = 0$.

For simulations in which the mutation rate to resistance is non-zero we quantify the emergence of resistance in the following way. For each simulation run we record the maximum density of the resistant strain. Runs in which this density reaches a level high enough to cause symptoms (a density of 300 in this case) are deemed to be infections in which resistance has emerged. The probability of resistance emergence is quantified as the fraction of runs in which this threshold level is reached.

Figure S6 shows results where the maximum tolerable drug concentration c_U causes significant suppression of the resistant strain. We stress however that if this were true then, by definition, the resistant strain is not really HLR and thus there really is no resistance problem to begin with. We include this extreme example as a benchmark against which comparisons can be made. Not surprisingly, in this case the conventional, high-dose, strategy best contains resistance emergence.

Figure S6. Example where conventional strategy of high-dose chemotherapy best prevents the emergence of resistance. (a) The dose-response curves for the wild type in blue ($r(c) = 0.00255(1 - \tanh(15(c - 0.3)))$) and the resistant strain in red ($r_m(c) = 0.0025(1 - \tanh(15(c - 0.45)))$) as well as the therapeutic window in green. Red dots indicate the probability of resistance emergence. Probability of resistance emergence is defined as the fraction of 1000 simulations for which resistance reached a density of at least 300 (and thus caused disease). (b) and (c) wild type density (blue), resistant density (red), and resource density (black) during infection for 20 representative realizations of a stochastic implementation of the model. (b) treatment at the smallest effective dose c_L, (c) treatment at the maximum tolerable dose c_U. Parameter values: $P(0) = 2$, $P_m(0) = 0$, $R(0) = 2000$, $\theta = 200$, $\delta = 0.1$, $d = 2$, $d_m = 2.7$, and $\mu = 10^{-2}$.

On the other hand, Figure S7 shows results where the maximum tolerable drug concentration c_U is not sufficient to directly suppress the resistant strain. As as result, from a clinical standpoint the drug is largely ineffective against the resistant strain. As can be seen, this seemingly small change from the results in Figure S6 reverses the prediction. Now the smallest effective dose best contains resistance emergence.

Figure S7. Example where a low-dose strategy best prevents the emergence of resistance. (a) The dose-response curves for the wild type in blue ($r(c) = 0.00255(1-\tanh(15(c-0.3)))$) and the resistant strain in red ($r_m(c) = 0.0025(1-\tanh(15(c-0.6)))$) as well as the therapeutic window in green. Red dots indicate the probability of resistance emergence. Probability of resistance emergence is defined as the fraction of 1000 simulations for which resistance reached a density of at least 300 (and thus caused disease). (b) and (c) wild type density (blue), resistant density (red), and resource density (black) during infection for 20 representative realizations of a stochastic implementation of the model. (b) treatment at the smallest effective dose c_L, (c) treatment at the maximum tolerable dose c_U. Parameter values: $P(0) = 2$, $P_m(0) = 0$, $R(0) = 2000$, $\theta = 200$, $\delta = 0.1$, $d = 2$, $d_m = 2.7$, and $\mu = 10^{-2}$.

Appendix 6 - Generalizing the pharmacokinetics

Here we illustrate how the qualitative conclusions of the main text hold more broadly by deriving the analogue of equation (4) for quite general forms of pharmacokinetics. For simplicity we will ignore the possibility that resistant strains might be present at the start of treatment.

For the sake of illustration we suppose that the drug is administered in some arbitrary way for a period of time of length T and then treatment is stopped. The question we ask is, how does increasing the duration of treatment T affect the probability of resistance emergence? More generally we might alter other aspects of treatment like dose size, inter-dose interval, etc but our focus on T will be sufficient to see how one would deal with these other factors as well.

To allow for more general pharmacokinetics we must model the dynamics of drug concentration explicitly. Once treatment has begun the model becomes

$$\frac{dp}{dt} = f(p, x, c) \quad \text{(6-1a)}$$

$$\frac{dx}{dt} = g(p, x, c) \quad \text{(6-1b)}$$

$$\frac{dc}{dt} = h(p, x, c, t) \quad \text{(6-1c)}$$

The third equation accounts for the pharmacokinetics of the drug and allows for the treatment protocol to vary through time. These equations must also be supplemented with an initial condition specifying the values of the variables at the start of treatment.

After time T has elapsed treatment is stopped and the dynamics then follow a different set of equations given by

$$\frac{d\tilde{p}}{dt} = \tilde{f}(\tilde{p}, \tilde{x}, \tilde{c}) \tag{6-2a}$$
$$\frac{d\tilde{x}}{dt} = \tilde{g}(\tilde{p}, \tilde{x}, \tilde{c}) \tag{6-2b}$$
$$\frac{d\tilde{c}}{dt} = \tilde{h}(\tilde{p}, \tilde{x}, \tilde{c}, t) \tag{6-2c}$$

The tildes reflect the fact that the functional form of the dynamical system might change when treatment is stopped (e.g., there is no longer any input of the drug in the function $\tilde{h}$ as compared with the function h), and thus the variables follow a different trajectory than they would have under treatment. This system of differential equation must also be supplemented with an initial condition as well, and this requires $\tilde{p}(T) = p(T)$, $\tilde{x}(T) = x(T)$, and $\tilde{c}(T) = c(T)$. Notice that the trajectories of the new variables $\tilde{p}, \tilde{x}$ and $\tilde{c}$ therefore depend on the duration of treatment T because this duration will affect their initial values.

With the above formalism we can write the hazard as

$$H(T) = \int_0^T \lambda\pi \mathrm{d}s + \int_T^\infty \tilde{\lambda}\tilde{\pi}\mathrm{d}s \tag{6-3}$$

where we have simplified the notation by using a tilde above a function to indicate that the function is evaluated along the variables with a tilde. Differentiating with respect to T gives

$$\frac{dH}{dT} = \lambda\pi|_{s=T} - \tilde{\lambda}\tilde{\pi}|_{s=T} + \int_T^\infty \frac{d}{dT}\tilde{\lambda}\tilde{\pi}\mathrm{d}s \tag{6-4}$$

By the continuity of the state variables the first two terms cancel and therefore we have

$$\frac{dH}{dT} = \int_T^\infty \frac{d}{dT}\tilde{\lambda}\tilde{\pi}\mathrm{d}s \tag{6-5}$$

Now $\tilde{\lambda}$ and $\tilde{\pi}$ depend on T because they depend on the trajectories of the variables $\tilde{p}, \tilde{x}$ and $\tilde{c}$, and the trajectories of these variables in turn depend on their initial conditions (which depend on T as described above). We can capture this notationally by treating the variables $\tilde{p}, \tilde{x}$ and $\tilde{c}$ as functions of T. Thus we have

$$\begin{aligned}\frac{dH}{dT} &= \int_T^\infty \frac{d}{dT}\tilde{\lambda}\tilde{\pi}\mathrm{d}s \\ &= \int_T^\infty \pi\left(\frac{\partial\lambda}{\partial\tilde{p}}\frac{\partial\tilde{p}}{\partial T} + \frac{\partial\lambda}{\partial\tilde{c}}\frac{\partial\tilde{c}}{\partial T}\right) + \lambda\left(\nabla_{\tilde{x}}\pi \cdot \tilde{x}_T + \frac{\partial\pi}{\partial\tilde{c}}\frac{\partial\tilde{c}}{\partial T}\right)\mathrm{d}s\end{aligned}$$

We can see that this has a form that is identical to *de novo* part of equation (4) except that now the drug concentration is no longer directly under our control. Instead, changes in T affect resistance emergence by how they affect changes in drug concentration. More generally, the very same potentially opposing processes as those in equation 4 will arise regardless of how we alter the drug dosing regimen because any such alteration must ultimately be mediated through its affect on the drug concentration at each point in time during an infection.

References

[1] Zhao X, Drlica K. Restricting the selection of antibiotic-resistant mutants: a general strategy derived from fluoroquinolone studies. Clinical Infectious Diseases. 2001;33:S147–S156.

[2] Zhao X, Drlica K. Restricting the selection of antibiotic-resistant mutant bacteria: measurement and potential use of the mutant selection window. The Journal of Infectious Diseases. 2002;185:561–565.

[3] Drlica K, Zhao X. Mutant selection window hypothesis updated. Clinical Infectious Diseases. 2007;44:681–688.

[4] Olofsson SK, Cars O. Optimizing drug exposure to minimize selection of antibiotic resistance. Clinical Infectious Diseases. 2007;45:S129–S136.

[5] Mouton JW, Ambrose PG, Canton R, Drusano GL, Harbarth S, MacGowan A, et al. Conserving antibiotics for the future: new ways to use old and new drugs from a pharmacokinetic and pharmacodynamic perspective. Drug Resistance Updates. 2011;14:107–117.

[6] Hastings I. Why we should effectively treat malaria. Trends in Parasitology. 2011;27:51–52.

[7] Canton R, Morosini MI. Emergence and spread of antibiotic resistance following exposure to antibiotics. FEMS Microbiology Reviews. 2011;35:977–991.

[8] Ankomah P, Levin BR. Exploring the collaboration between antibiotics and the immune response in the treatment of acute, self-limiting infections. Proceedings of the National Academy of Science. 2014;111:8331–8338.

[9] Huijben S, Chan BHK, Read AF. Relevance of undetectably rare resistant malaria parasites in treatment failure: experimental evidence from *Plasmodium chabaudi*. American Journal of Hygiene and Tropical Medicine. 2015;92:1214–1221.
