# Supplementary figures and images for: Does High-Dose Antimicrobial Chemotherapy Prevent the Evolution of Resistance?
Source: PLoS Comput Biol. 2016 Jan 28;12(1):e1004689. doi: 10.1371/journal.pcbi.1004689 (PMC4731197; doi:10.1371/journal.pcbi.1004689)

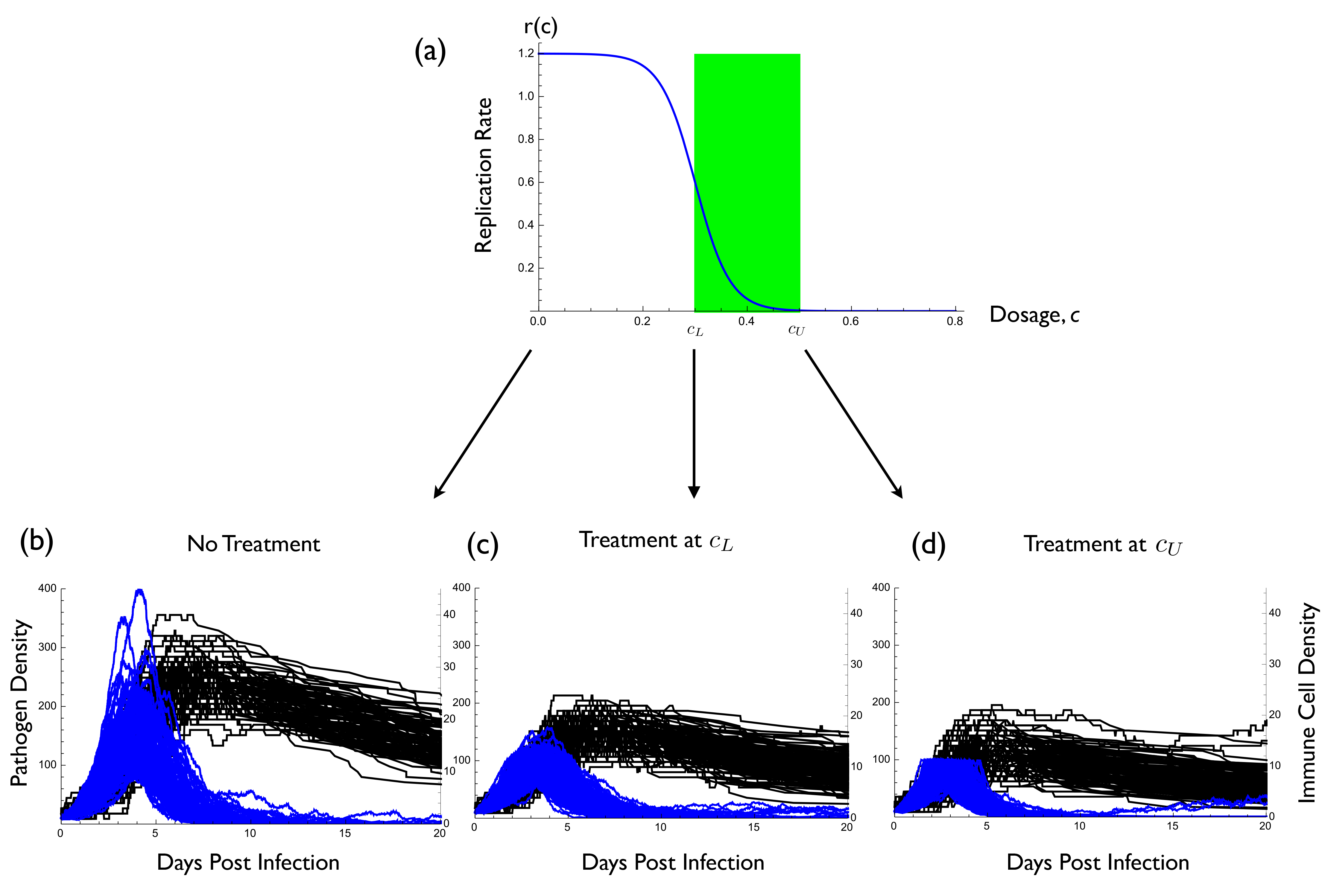

Supplement: S1 Fig — (a) The dose-response curve r(c) = 0.6(1−tanh(15(c−0.3))) as well as the therapeutic window in green. (b), (c) and (d) show wild type pathogen density (blue) and immune molecule density (black) during infection for 1000 representative realizations of a stochastic implementation of the model. (b) no treatment, (c) treatment at the smallest effective dose c L, (d) treatment at the maximum tolerable dose c U. Parameter values are P(0) = 10, I(0) = 2, α = 0.05, δ = 0.05, κ = 0.075, μ = 0, and γ = 0.01. (TIF) [file pcbi.1004689.s002.tif]

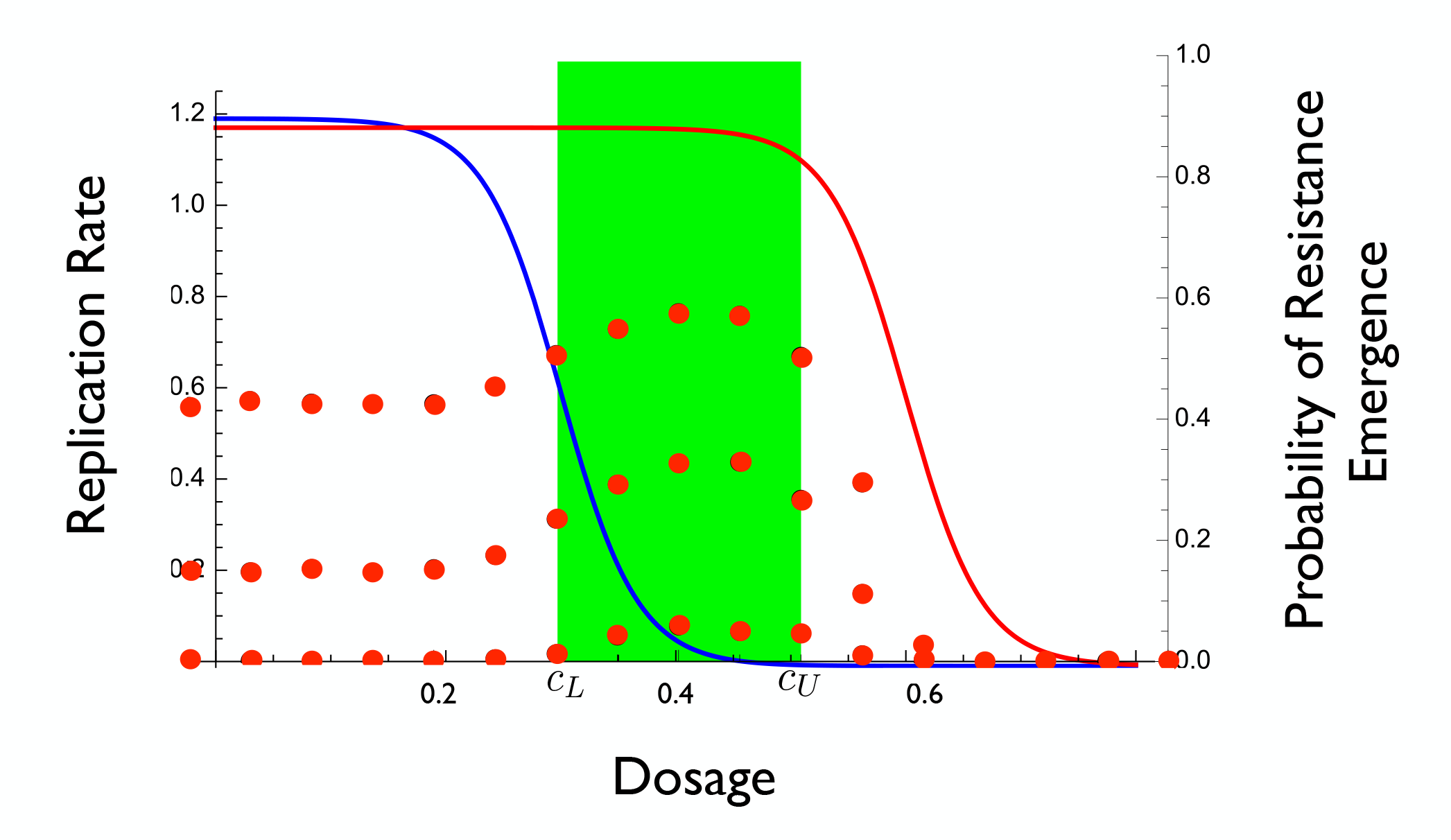

Supplement: S2 Fig — Simulation is identical to that for Fig 3a except for the initial conditions. The dose-response curves for the wild type in blue (r(c) = 0.6(1−tanh(15(c−0.3)))) and the resistant strain in red (r m(c) = 0.59(1−tanh(15(c−0.6)))) as well as the therapeutic window in green. Red dots indicate the probability of resistance emergence, and for three different initial conditions. Probability of resistance emergence is defined as the fraction of 5000 simulations for which resistance reached a density of at least 100 (and thus caused disease). Top set of dots have P(0) = 5, P m(0) = 5; middle set of dots have P(0) = 7, P m(0) = 3; bottom set of dots have P(0) = 10, P m(0) = 0. Other parameter values are I(0) = 2, α = 0.05, δ = 0.05, κ = 0.075, μ = 10−2, and γ = 0.01. (TIF) [file pcbi.1004689.s003.tif]

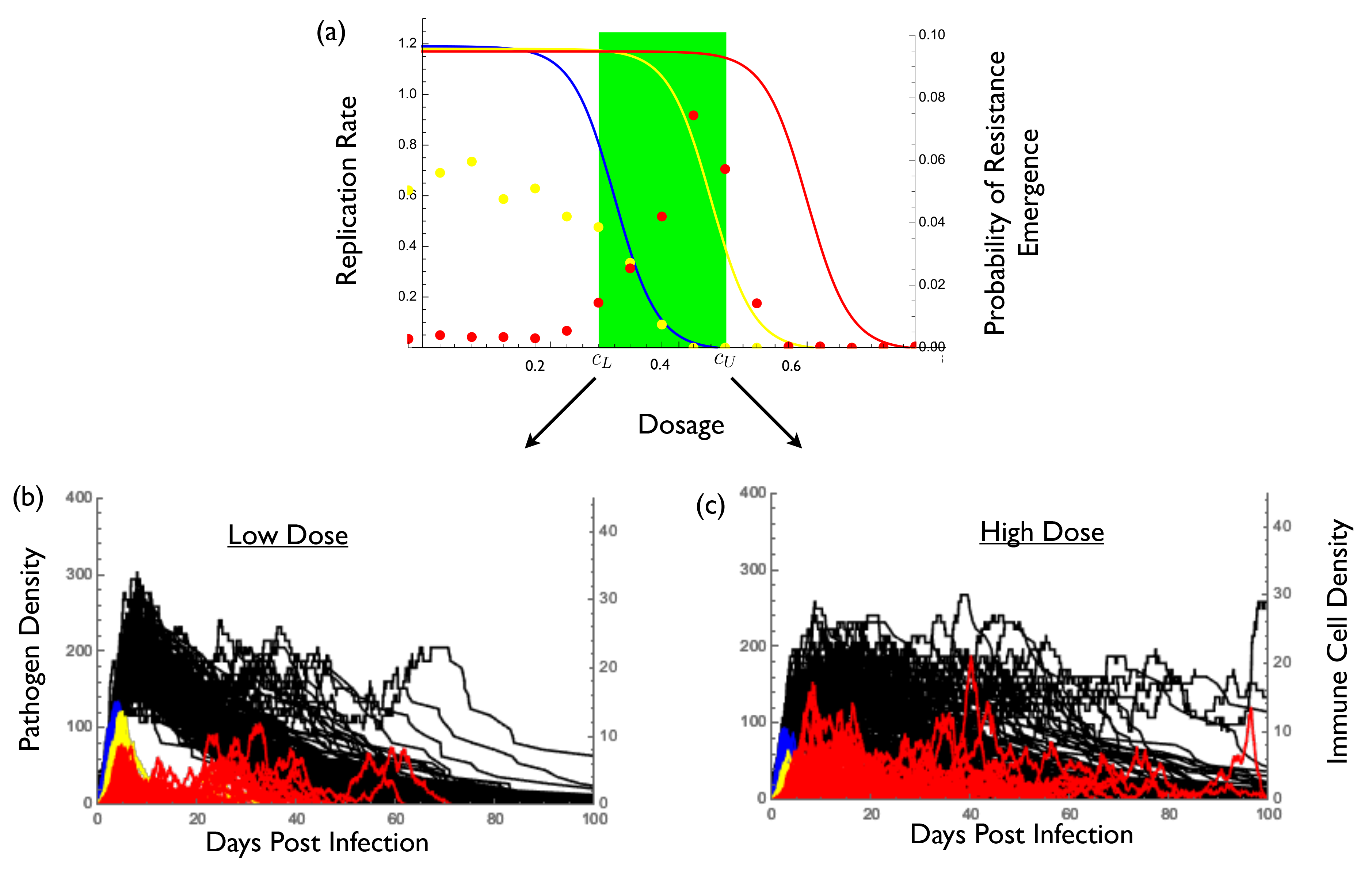

Supplement: S3 Fig — (a) The dose-response curves for the wild type in blue (r(c) = 0.6(1−tanh(15(c−0.3)))), the intermediate strain in yellow (r m2(c) = 0.595(1−tanh(15(c−0.45)))), and the HLR strain in red (r m2(c) = 0.59(1−tanh(15(c−0.6)))) as well as the therapeutic window in green. Dots indicate the probability of emergence for the intermediate strain (yellow) and the HLR strain (red). Probability of emergence is defined as the fraction of 5000 simulations for which the strain reached a density of at least 100. (b) and (c) wild type density (blue), intermediate strain density (yellow), HLR strain density (red), and immune molecule density (black) during infection for 1000 representative realizations of a stochastic implementation of the model. (b) treatment at the smallest effective dose c L, (c) treatment at the maximum tolerable dose c U. Parameter values are P(0) = 10, P m1(0) = 0, P m2(0) = 0, I(0) = 2, α = 0.05, δ = 0.05, κ = 0.075, μ = 10−2, μ 1 = 10−2, and γ = γ m1 = γ m2 = 0.01. (TIF) [file pcbi.1004689.s004.tif]

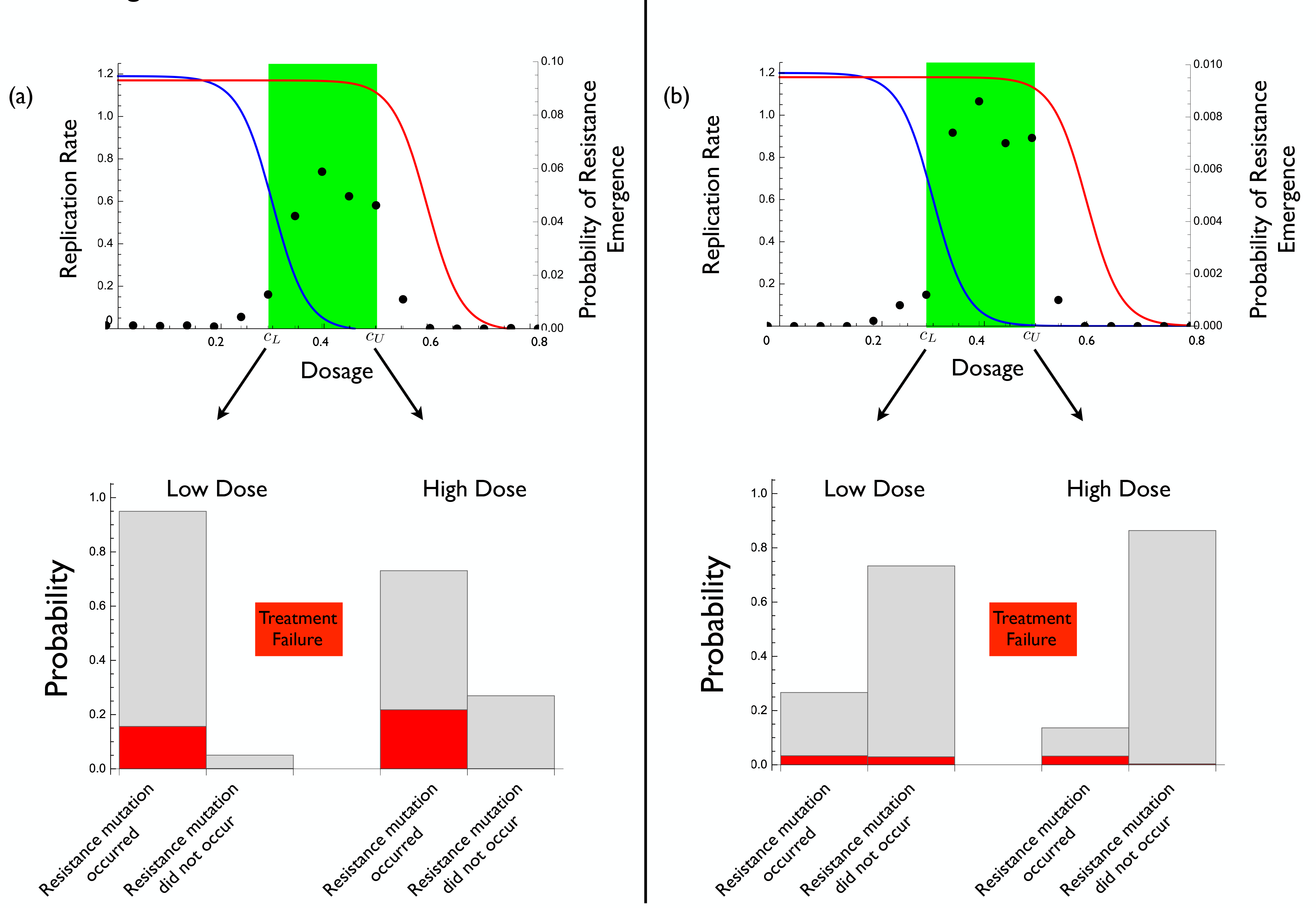

Supplement: S4 Fig — (a) The dose-response curves for the wild type in blue (r(c) = 0.6(1−tanh(15(c−0.3)))) and the resistant strain in red (r m(c) = 0.59(1−tanh(15(c−0.6)))) as well as the therapeutic window in green. Dots indicate the probability of resistance emergence. Probability of resistance emergence is defined as the fraction of 5000 simulations for which resistance reached a density of at least 100 (and thus caused disease). Parameter values are P(0) = 10, I(0) = 2, α = 0.05, δ = 0.05, κ = 0.075, μ = 10−2, and γ = 0.01. Bar graphs: the probability that a resistant strain appears by mutation is indicated by the left-hand grey bars for each drug concentration (the right-hand grey bar is the probability that a resistant strain does not appear). The probability of treatment failure for a specific drug dose is the sum of the red bars for that dose. (b) Same as panel (a) but with mutation rate decreased to μ = 10−3. (TIF) [file pcbi.1004689.s005.tif]

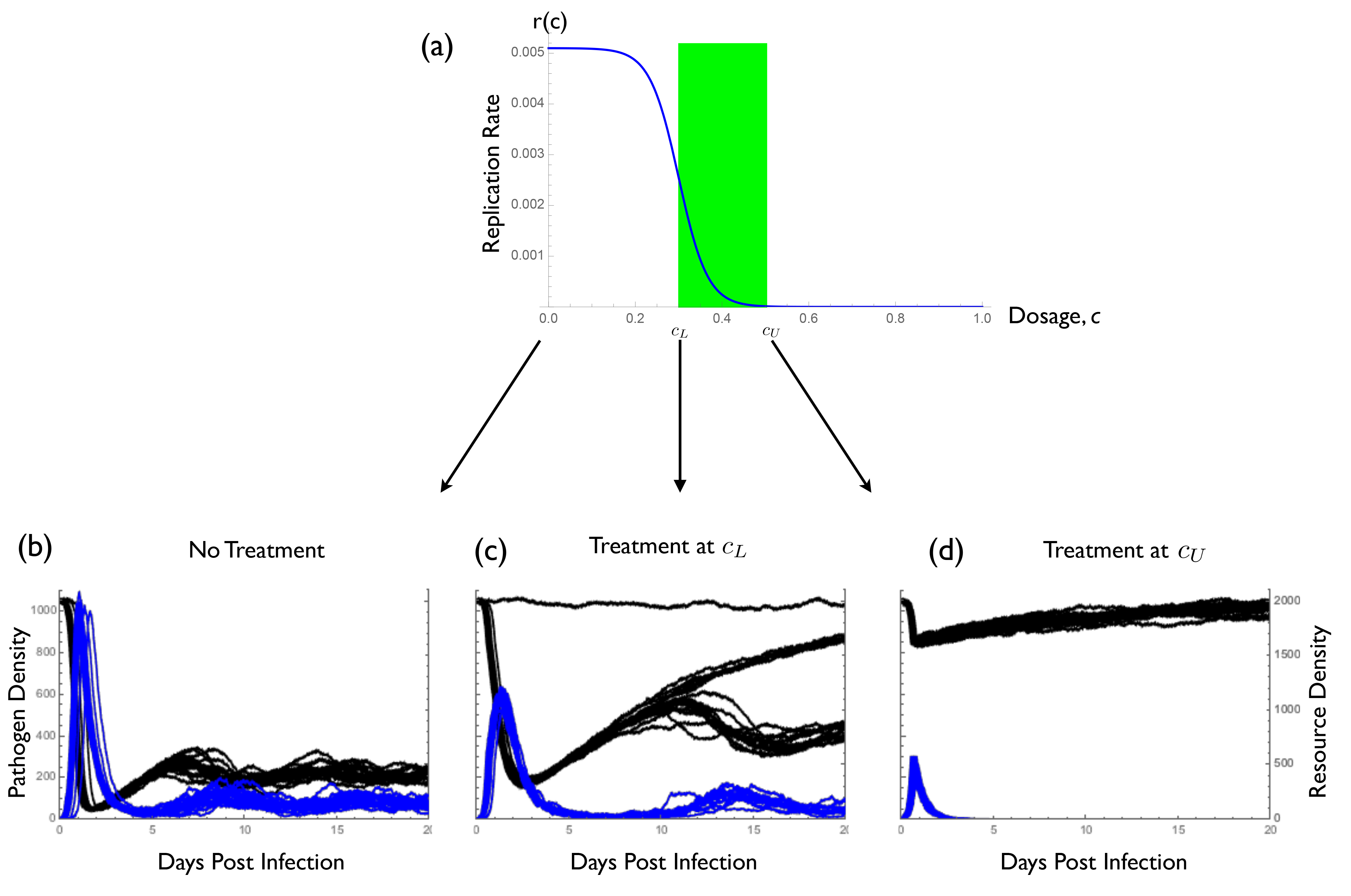

Supplement: S5 Fig — (a) The dose-response curve r(c) = 0.00255(1−tanh(15(c−0.3))) as well as the therapeutic window in green. (b), (c) and (d) show wild type pathogen density (blue) and resource density (black) during infection for 20 representative realizations of a stochastic implementation of the model. (b) no treatment, (c) treatment at the smallest effective dose c L, (d) treatment at the maximum tolerable dose c U. Parameter values are P(0) = 2, R(0) = 2000, θ = 200, δ = 0.1, d = 2, and μ = 0. (TIF) [file pcbi.1004689.s006.tif]

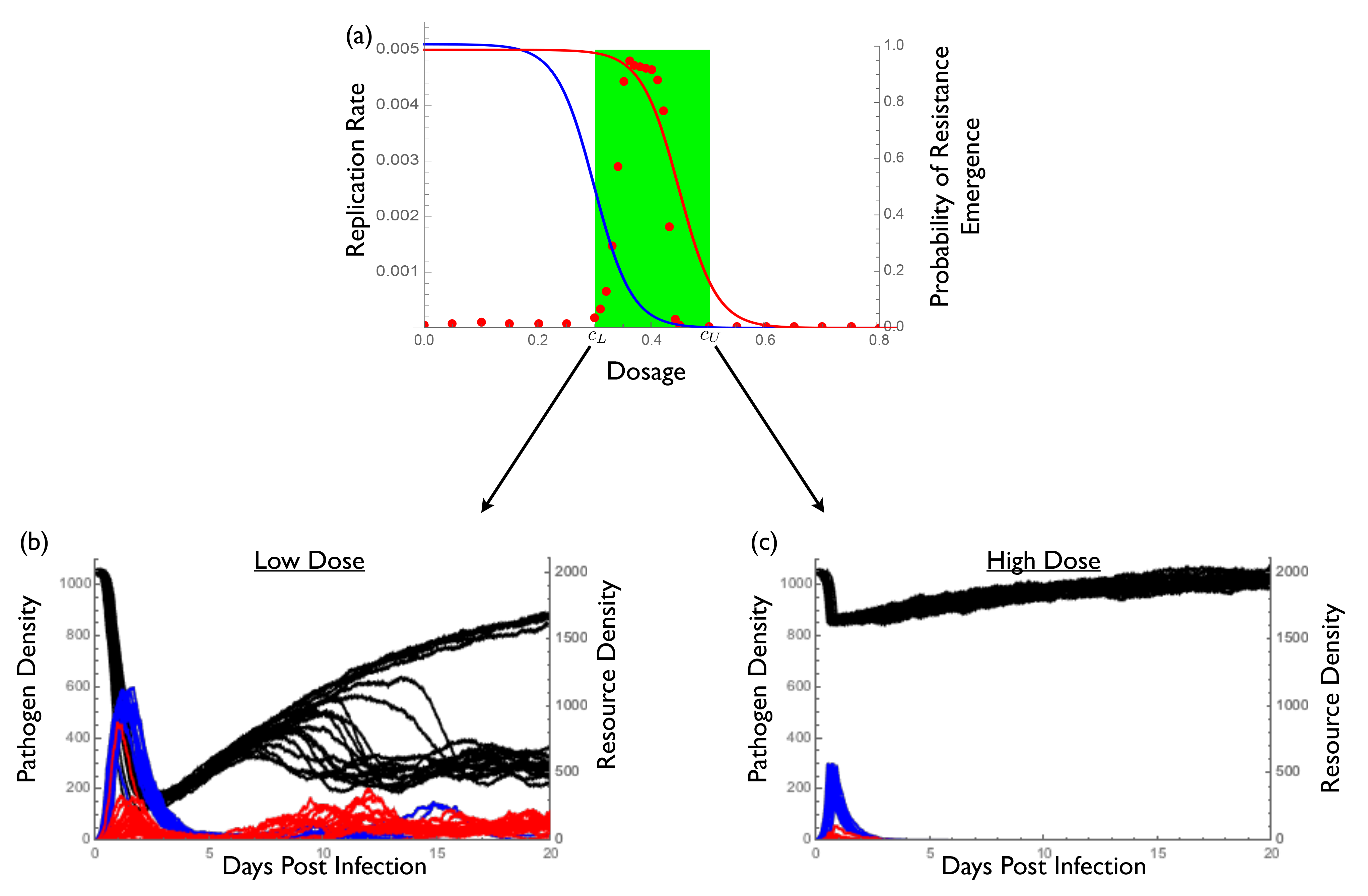

Supplement: S6 Fig — (a) The dose-response curves for the wild type in blue (r(c) = 0.00255(1−tanh(15(c−0.3)))) and the resistant strain in red (r m(c) = 0.0025(1−tanh(15(c−0.45)))) as well as the therapeutic window in green. Red dots indicate the probability of resistance emergence. Probability of resistance emergence is defined as the fraction of 1000 simulations for which resistance reached a density of at least 300 (and thus caused disease). (b) and (c) wild type density (blue), resistant density (red), and resource density (black) during infection for 20 representative realizations of a stochastic implementation of the model. (b) treatment at the smallest effective dose c L, (c) treatment at the maximum tolerable dose c U. Parameter values: P(0) = 2, P m(0) = 0, R(0) = 2000, θ = 200, δ = 0.1, d = 2, d m = 2.7, and μ = 10−2. (TIF) [file pcbi.1004689.s007.tif]

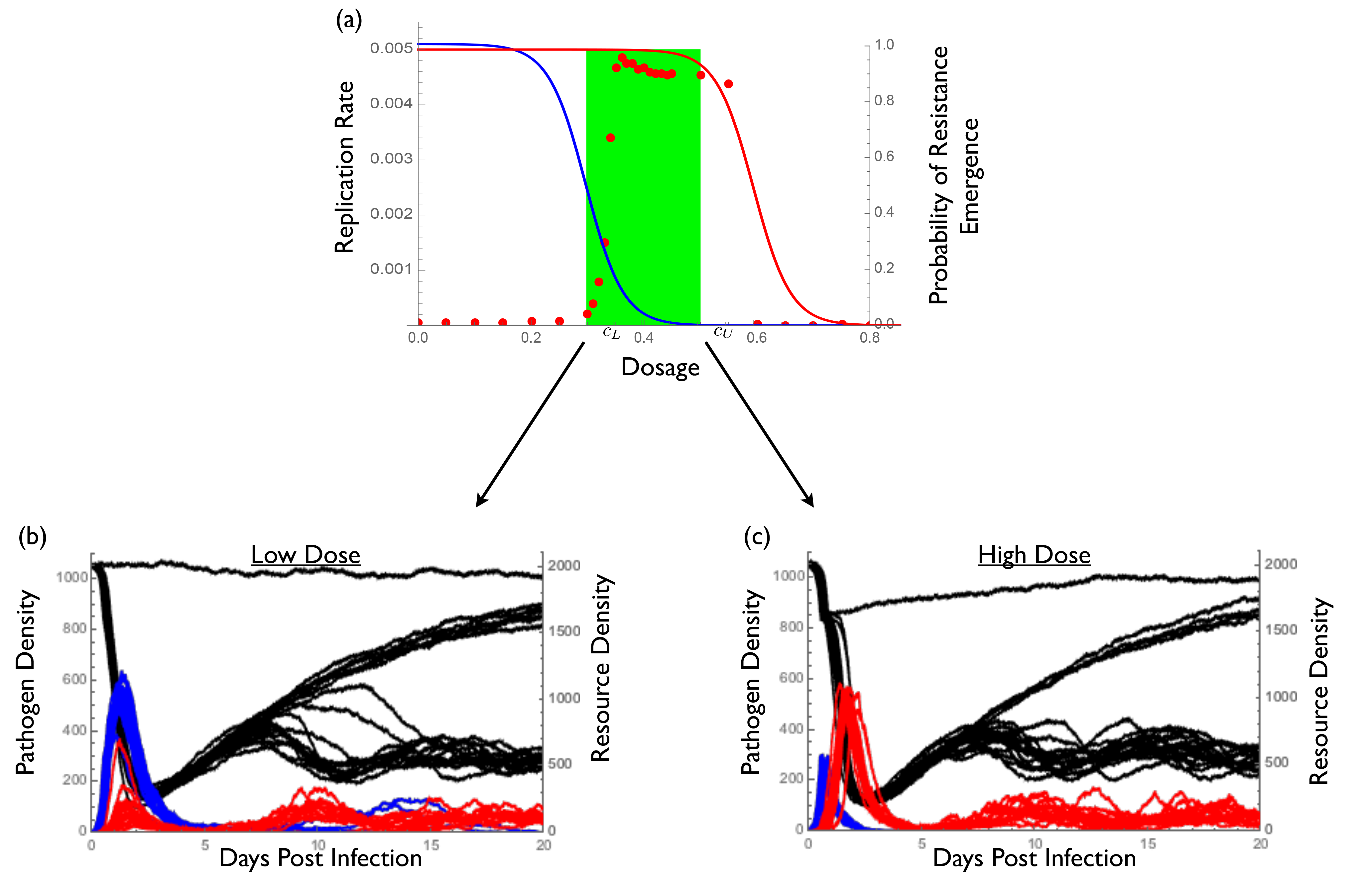

Supplement: S7 Fig — (a) The dose-response curves for the wild type in blue (r(c) = 0.00255(1−tanh(15(c−0.3)))) and the resistant strain in red (r m(c) = 0.0025(1−tanh(15(c−0.6)))) as well as the therapeutic window in green. Red dots indicate the probability of resistance emergence. Probability of resistance emergence is defined as the fraction of 1000 simulations for which resistance reached a density of at least 300 (and thus caused disease). (b) and (c) wild type density (blue), resistant density (red), and resource density (black) during infection for 20 representative realizations of a stochastic implementation of the model. (b) treatment at the smallest effective dose c L, (c) treatment at the maximum tolerable dose c U. Parameter values: P(0) = 2, P m(0) = 0, R(0) = 2000, θ = 200, δ = 0.1, d = 2, d m = 2.7, and μ = 10−2. (TIF) [file pcbi.1004689.s008.tif]
